# Bioactive-Guided Phytochemical Investigations, In Vitro and In Silico Alpha-Glucosidase Inhibition of Two Vietnamese Medicinal Plants *Dicranopteris linearis* and *Psychotria adenophylla*

**DOI:** 10.3390/ph16091253

**Published:** 2023-09-05

**Authors:** Thuc-Huy Duong, Y Thien Vu, Nguyen Phuoc Long, Nguyen-Hong-Nhi Phan, Nguyen-Kim-Tuyen Pham, Jirapast Sichaem, Nguyen-Khanh-Duy Kieu, Chi-Bao Duong, Thanh-Trung Nguyen, Van-Son Dang, Huy Truong Nguyen

**Affiliations:** 1 Department of Chemistry, University of Education, 280 An Duong Vuong Street, District 5, Ho Chi Minh City 700000, Vietnam; huydt@hcmue.edu.vn (T.-H.D.); hongnhi1697@gmail.com (N.-H.-N.P.); kieunguyenkhanhduy2001@gmail.com (N.-K.-D.K.); duongchibao.sphoaa@gmail.com (C.-B.D.); 2 Faculty of Pharmacy, Ton Duc Thang University, Ho Chi Minh City 700000, Vietnam; vuthieny@tdtu.edu.vn; 3 Department of Pharmacology and PharmacoGenomics Research Center, Inje University College of Medicine, Busan 47392, Republic of Korea; phuoclong@inje.ac.kr; 4 Faculty of Environment, Sai Gon University, 273 An Duong Vuong, Ward 3, District 5, Ho Chi Minh City 700000, Vietnam; phngktuyen@sgu.edu.vn; 5 Research Unit in Natural Products Chemistry and Bioactivities, Faculty of Science and Technology, Thammasat University Lampang Campus, Lampang 52190, Thailand; jirapast@tu.ac.th; 6 Institute of Research and Development, Duy Tan University, Danang 550000, Vietnam; 7 Center for Pharmaceutical Biotechnology, School of Medicine and Pharmacy, Duy Tan University, Danang 550000, Vietnam; 8 Vietnam Academy of Science and Technology, Graduate University of Science and Technology, 18 Hoang Quoc Viet, Cau Giay, Hanoi 100000, Vietnam; dvsonitb@gmail.com; 9 Institute of Tropical Biology, Vietnam Academy of Science and Technology, 85 Tran Quoc Toan Street, District 3, Ho Chi Minh City 700000, Vietnam

**Keywords:** *Dicranopteris linearis*, *Psychotria adenophylla*, alpha-glucosidase, flavonoids, molecular docking

## Abstract

Little is known about the chemical and biological profiles of *Dicranopteris linearis* and *Psychotria adenophylla*. No previous studies have investigated alpha-glucosidase inhibition using extracts from *D. linearis* and *P. adenophylla*. In this paper, bioactive-guided isolation procedures were applied to the plants *D. linearis* and *P. adenophylla* based on alpha-glucosidase inhibition. From the most active fractions, 20 compounds (**DL1**–**DL13** and **PA1**–**PA7**) were isolated. The chemical structures were elucidated using spectroscopic data and compared with those available in the literature. These compounds were evaluated for alpha-glucosidase inhibition, while a molecular docking study was performed to elucidate the mechanisms involved. Consequently, *D. linearis* and *P. adenophylla* might serve as a good potential for developing new antidiabetic preparations.

## 1. Introduction

*Dicranopteris linearis* (Burm. F.) Underw. Is a common fern belonging to the Gleicheniaceae family. This species is widely distributed in Africa and Asia, especially in the dry mountainous regions [1]. The extracts of *D. linearis* showed anticancer, antibacterial, antioxidant, analgesic, and anti-HIV activities [1,2,3,4,5]. This plant is commonly used as a folk medicine to treat fever (Malaysia), intestinal worms (Indochina), asthma, infertility in women (India), wounds (Papua New Guinea) [6], cough, allergies, and respiratory disorders (Mymensingh) [7]. Until now, 21 compounds have been isolated from *D. linearis* [1,2,3,4,5]. The major components are glycosides with aglycone parts: diterpenes, flavanols, and monoaromatic compounds. *D. linearis* is native to Vietnam, but alpha-glucosidase inhibition of its extracts and compounds derived from it has not been studied yet.

The genus *Psychotria* (Rubiaceae) comprises approximately 1700 species, popularly distributed in tropical and subtropical areas [8]. Different parts of these species (leaves, roots, and rhizomes) have been traditionally used to treat fever, bronchitis, ulcers, stomachaches, and gynecological hemorrhage in females [9]. Pharmacological studies have indicated that *Psychotria* plants exhibit various biological activities, including antimicrobial, antiviral, analgesic, hypoglycemic, and strong cytotoxic activities against several cancer cell lines [10,11]. The chemical data of the genus *Psychotria* have been comprehensively studied, indicating that the major compounds of this genus are alkaloids and terpenoids. Some alkaloids are the biomarkers of *Psychotria* plants [12]. *Psychotria adenophylla* wall is distributed in the south of Vietnam, and the phytochemical data on this plant are scarce. There has been only one report about the chemical constituents of *P. adenophylla* growing in India, which indicated the presence of eight sterols and triterpenes, including *β*-sitosterol, betulin, betulinic acid, *α*-amyrin, ursolic acid, friedelin, bauerenol, and bauerenol acetate [13].

**Figure 1:**
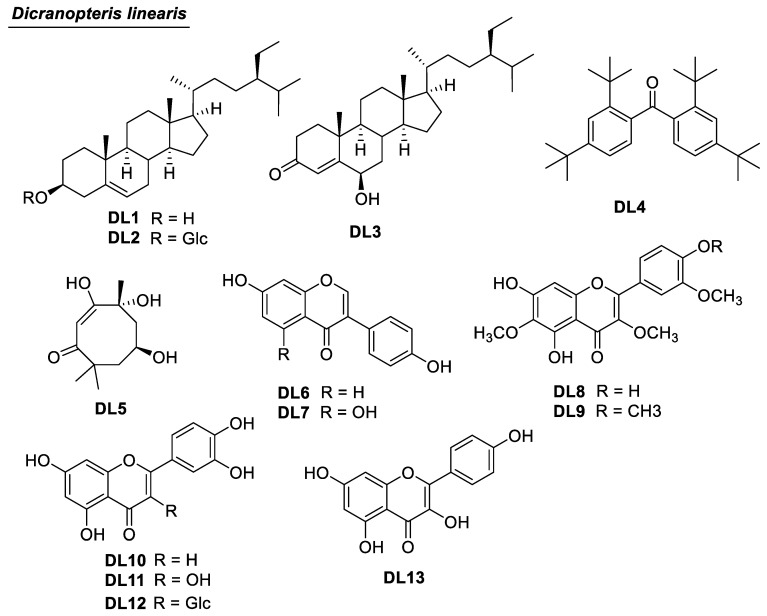
Chemical structures of compounds **DL1**–**DL13** from *D. linearis.*

**Figure 2:**
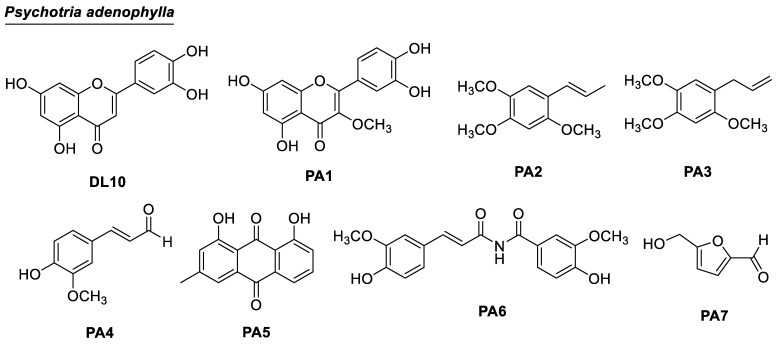
Chemical structures of compounds **DL10** and **PA1**–**PA7** from *P. adenophylla.*

There is limited information available concerning the chemical and biological profiles of *D. linearis* and *P. adenophylla*. Additionally, there have been no prior investigations into the potential of extracts from these plants to inhibit alpha-glucosidase. During our systematic research on alpha-glucosidase inhibitors from Vietnamese medicinal plants, bioactive-guided isolation procedures were applied to the plants *D. linearis* and *P. adenophylla* based on alpha-glucosidase inhibition. Twenty compounds (**DL1**–**DL13** and **PA1**–**PA7**) were isolated from the most active fraction (Figure 1 and Figure 2). The chemical structures were elucidated using spectroscopic data and compared with those available in the literature. These compounds were evaluated for alpha-glucosidase inhibition, and a molecular docking study was performed to elucidate the mechanisms involved.

## 2. Results

Extracts/fractions from *D. linearis* and *P. adenophylla* were evaluated for alpha-glucosidase inhibition (Table 1). The most bioactive extract of each plant was selected for further isolation.

### 2.1. Phytochemical Identification and Alpha-Glucosidase Inhibition of Isolated Compounds of D. linearis

**Figure 3:**
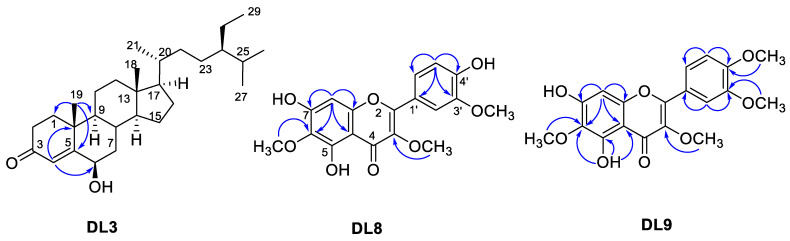
Selected HMBC correlations of compounds **DL3**, **DL8**, and **DL9** from *P. adenophylla.*

Thirteen compounds were isolated from *D. linearis.* They were structurally elucidated as *β*-sitosterol (**DL1**) [14], daucosterol (**DL2**) [15], 6-hydroxystigmast-4-en-3-one (**DL3**) [16], 2,2′,4,4′-tetra-*tert*-butylbenzophenone (**DL4**) [17], chakyunglupulin B (**DL5**) [18], daidzein (**DL6**) [19], genistein (**DL7**) [20], jaceidin (**DL8**) [21], bonanzin (**DL9**) [22], luteolin (**DL10**) [23], quercetin (**DL11**) [24], isoquercetin (**DL12**) [25], and kaempferol (**DL13**) [26]. The chemical structures of all Isolated compounds were determined using 1D and 2D NMR methods. For compound **DL3**, the position of 6-OH was defined using the Heteronuclear Multiple Bond Correlation (HMBC) of H-4 to C-6 (Figure 3). The configuration of C-6 was defined based on the small *J* value of H-6 [16]. The positions of the methoxy groups of multi-oxygenated flavonoids **DL8** and **DL9** were defined using HMBC correlations (Figure 3).

The physicochemical properties of isolated compounds are described below.

*β*-Sitosterol (**DL1**). White amorphous powder. ^1^H-NMR (500 MHz, acetone-*d*_6_) and ^13^C-NMR (125 MHz, acetone-*d*_6_) data were consistent with those reported in the literature [14].

Daucosterol (**DL2**). White amorphous powder. ^1^H-NMR (500 MHz, DMSO-*d*_6_) and ^13^C-NMR (125 MHz, DMSO-*d*_6_) data were consistent with those reported in the literature [15].

6-Hydroxystigmast-4-en-3-one (**DL3**). White amorphous powder. ^1^H-NMR (500 MHz, chloroform-*d*, *δ* ppm, *J* in Hertz): 5.82 (s, H-4), 4.35 (t, *J =* 2.0 Hz, H-6), 1.38 (s, H-19), 0.92 (d, *J =* 6.5 Hz, H-21), 0.84 (overlap, H-29), 0.83 (overlap, H-27), 0.81 (d, *J =* 7.0 Hz, H-26), 0.74 (s, H-18). ^13^C NMR (chloroform-*d*, 125 MHz, *δ* ppm):-200.6 (C-3), 168.6 (C-5), 126.5 (C-4), 73.5 (C-6), 56.2 (C-17), 56.0 (C-14), 53.8 (C-9), 46.0 (C-24), 42.7 (C-13), 39.8 (C-12), 38.7 (C-7), 38.1 (C-10), 37.3 (C-1), 36.3 (C-20), 34.4 (C-2), 34.1 (C-22), 29.9 (C-8), 29.3 (C-25), 28.3 (C-16), 26.3 (C-23), 24.3 (C-15), 23.2 (C-28), 21.1 (C-11), 20.0 (C-27), 19.7 (C-19), 19.2 (C-26), 18.9 (C-21), 12.2 (C-18), and 12.1 (C-29). These data were consistent with those reported in the literature [16].

2,2′,4,4′-Tetra-*tert*-butylbenzophenone (**DL4**). Colorless oil. ^1^H-NMR (500 MHz, chloroform-*d*, *δ* ppm, *J* in Hertz): 7.53 (*d*, *J =* 9.0 Hz, H-6), 7.35 (*t*, *J =* 2.3 Hz, H-4), 7.12 (*d*, *J =* 8.5, 2.5 Hz, H-5), 1.33 (*s*, H-10), 1.28 (*s*, H-9). ^13^C NMR (125 MHz, chloroform-*d*, *δ* ppm): 196.8 (C=O), 147.2 (C-1), 141.1 (C-2), 138.5 (C-3), 124.6 (C-4), 124.1 (C-5), 119.3 (C-6), 35.8 (C-7), 35.0 (C-8), 31.6 (C-9), and 30.3 (C-10). These data were consistent with those reported in the literature [17].

Chakyunglupulin B (**DL5**). Colorless oil. ^1^H-NMR (500 MHz, chloroform-*d*, *δ* ppm, *J* in Hertz): 5.69 (*s*, H-2), 4.33 (*m*, H-6), 2.46 (*dt*, *J = 14.0*, *2.8* Hz, H-5*β*), 1.97 (*dt*, *J =* 14.5, 2.8 Hz, H-7*β*), 1.78 (*overlap*, H-5α), 1.78 (*s*, H-11), 1.53 (*dd*, *J =* 14.5, 4.0 Hz, H-7α), 1.47 (*s*, H-9), 1.27 (*s*, H-10). ^13^C NMR (125 MHz, chloroform-*d*, *δ* ppm):182.5 (C-1), 171.2 (C-3), 113.1 (C-2), 86.8 (C-4), 67.0 (C-6), 47.5 (C-7), 45.8 (C-5), 36.1 (C-8), 30.8 (C-10), 27.2 (C-11), and 26.6 (C-9). These data were consistent with those reported in the literature [18].

Daidzein (**DL6**). Yellow amorphous powder. ^1^H-NMR (500 MHz, DMSO-*d*_6_, *δ* ppm, *J* in Hertz): 9.52 (*s*, 4′-OH), 8.29 (*s*, H-2), 7.97 (*d*, *J =* 8.5 Hz, H-5), 7.38 (*d*, *J =* 8.5 Hz, H-2′), 7.38 (*d*, *J =* 8.5 Hz, H-6′), 6.94 (*dd*, *J =* 8.5, 2.0 Hz, H-6), 6.87 (*d*, *J =* 2.5 Hz, H-8), 6.81 (*d*, *J =* 8.5 Hz, H-3′), 6.81 (*d*, *J =* 8.5 Hz, H-5′). ^13^C NMR (125 MHz, DMSO-*d*_6_, *δ* ppm): 174.6 (C-4), 162.3 (C-7), 157.4 (C-9), 157.4 (C-4′), 152.9 (C-2), 129.9 (C-2′), 129.9 (C-6′), 127.0 (C-5), 123.5 (C-3), 122.6 (C-1′), 116.6 (C-10), 115.1 (C-6), 114.9 (C-3′), 114.9 (C-5′), and 101.7 (C-8). These data were consistent with those reported in the literature [19].

Genistein (**DL7**). Yellow amorphous powder. ^1^H-NMR (500 MHz, Acetone-*d*_6_, *δ* ppm, *J* in Hertz): 13.03 (*s*, 5-OH), 8.16 (*s*, H-2), 7.45 (*d*, *J =* 8.5 Hz, H-2′), 7.45 (*d*, *J =* 8.5 Hz, H-6′), 6.90 (*d*, *J =* 8.5 Hz, H-3′), 6.90 (*d*, *J =* 8.5 Hz, H-3′), 6.42 (*d*, *J = 2*.5 Hz, H-8) and 6.28 (*d*, *J* = *2*.5 Hz, H-6). ^13^C NMR (125 MHz, Acetone-*d*_6_, *δ* ppm): 181.9 (C-4), 165.3 (C-7), 163.9 (C-5), 159.2 (C-9), 158.6 (C-4′), 154.3 (C-2), 131.2 (C-2′), 131.2 (C-6′), 124.3 (C-3), 123.8 (C-1′), 116.0 (C-3′), 116.0 (C-5′), 106.2 (C-10), 99.9 (C-6), and 94.5 (C-8). These data were consistent with those reported in the literature [20].

Jaceidin (**DL8**). Yellow amorphous powder. ^1^H-NMR (500 MHz, Acetone-*d*_6_, *δ* ppm, *J* in Hertz): 7.71 (*d*, *J =* 2.0, H-2′), 7.60 (*dd*, *J =* 9.0, 2.0, H-6′), 6.99 (*d*, *J =* 9.0, H-5′), 6.80 (*s*, H-8), 3.98 (*s*, 3′-OCH_3_), 3.87 (*s*, 6-OCH_3_) and 3.80 (*s*, 3-OCH_3_). ^13^C NMR (125 MHz, Acetone-*d*_6_, *δ* ppm): 177.3 (C-4), 160.1 (C-7), 156.8 (C-2), 153.2 (C-9), 151.8 (C-5), 149.3 (C-4′), 146.1 (C-3′), 136.0 (C-3), 133.2 (C-6), 122.9 (C-1′), 122.2 (C-6′), 116.4 (C-5′), 112.1 (C-2′), 107.1 (C-10), 97.1 (C-8), 60.5 (6-OCH_3_), 60.1 (3-OCH_3_), and 56.8 (3′-OCH_3_). These data were consistent with those reported in the literature [21].

Bonanzin (**DL9**). Yellow amorphous powder. ^1^H-NMR (500 MHz, Acetone-*d*_6_, *δ* ppm, *J* in Hertz): 12.7 (*s*, 5-OH), 7.71 (*dd*, *J =* 8.8, 2.3 Hz, H-6′), 7.66 (*d*, *J =* 2.0 Hz, H-2′), 7.13 (*d*, *J =* 8.5 Hz, H-5′), 6.85 (*s*, H-8), 3.99 (*s*, 4′-OCH_3_), 3.96 (*s*, 4′-OCH_3_), 3.89 (*s*, 3-OCH_3_) and 3.80 (*s*, 6-OCH_3_). ^13^C-NMR (125 MHz, Acetone-*d*_6_, *δ* ppm, *J* in Hertz: 180.7 (C-4), 160.3 (C-4′), 158.9 (C-7), 155.4 (C-9), 153.7 (C-5), 152.2 (C-2), 151.3 (C-3′), 139.3 (C-3), 133.2 (C-6), 124.2 (C-6′), 121.9 (C-1′), 115.8 (C-2′), 112.2 (C-5′), 107.1 (C-10), 91.8 (C-8), 60.6 (3-OCH_3_), 60.2 (6-OCH_3_), 56.9 (3′-OCH_3_), and 56.3 (4′-OCH_3_). These data were consistent with those reported in the literature [22].

Luteolin (**DL10**). Yellow amorphous powder. ^1^H-NMR (500 MHz, acetone-*d*_6_) and ^13^C-NMR (125 MHz, acetone-*d*_6_). These data were consistent with those reported in the literature [23].

Quercetin (**DL11**). Yellow amorphous powder. ^1^H-NMR (500 MHz, acetone-*d*_6_) and ^13^C-NMR (125 MHz, acetone-*d*_6_). These data were consistent with those reported in the literature [24].

Isoquercetin (**DL12**). Yellow powder. ^1^H-NMR (500 MHz, DMSO-*d*_6_) and ^13^C-NMR (125 MHz, DMSO-*d*_6_). These data were consistent with those reported in the literature [25].

Kaempferol (**DL13**). Yellow amorphous powder. ^1^H-NMR (500 MHz, acetone-*d*_6_) and ^13^C-NMR (125 MHz, acetone-*d*_6_). These data were consistent with those reported in the literature [26].

### 2.2. Phytochemical Identification and Alpha-Glucosidase Inhibition of Isolated Compounds of P. adenophylla

Eight compounds were isolated and structurally elucidated. They were luteolin (**DL10**) [23], 3-*O*-methylquercetin (**PA1**) [27], *α*-asarone (**PA2**) [28], *γ*-asarone (**PA3**) [29], coniferyl aldehyde (**PA4**) [30], chrysophanol (**PA5**) [31], tribulusimide D (**PA6**) [32], and 5-hydroxymethylfurfural (**PA7**) [33].

3-*O*-methylquercetin (**PA1**). Yellow amorphous powder. ^1^H-NMR (500 MHz, acetone-*d*_6_) and ^13^C-NMR (125 MHz, acetone-*d*_6_). These data were consistent with those reported in the literature [27].

*α*-Asarone (**PA2**). Colorless oil. ^1^H-NMR (500 MHz, acetone-*d*_6_, *δ* ppm, *J* in Hertz): 7.04 (1H, *s*, H-6), 6.67 (1H, *s*, H-3), 6.64 (1H, *dd*, 16.0, 2.0, H-1′), 6.13 (1H, *dq*, 16.0, 6.5, H-2′), 3.84 (3H, *s*, 2-OCH_3_), 3.82 (3H, *s*, 3-OCH_3_), 3.78 (3H, *s*, 5-OCH_3_), 1.84 (3H, *dd*, 6.5, 2.0, H-3′). ^13^C-NMR (125 MHz, acetone-*d*_6_, *δ* ppm): 151.1 (C-4), 149.6 (C-2), 143.7 (C-5), 125.3 (C-2′), 122.9 (C-1′), 118.3 (C-1), 110.7 (C-6), 98.5 (C-3), 56.3, 56.1, 55.7 (4-OCH_3_, 2-OCH_3_, and 5-OCH_3_ signals could be interchanged), and 17.8 (C-3′). These data were consistent with those reported in the literature [28].

*γ*-Asarone (**PA3**). Colorless oil. ^1^H-NMR (500 MHz, acetone-*d*_6_, *δ* ppm, *J* in Hertz): 6.76 (1H, *s*, H-6), 6.69 (1H, *s*, H-3), 5.95 (1H, *m*, H-2′), 5.02 (1H, *m*, H-3′a), 4.97 (1H, *m*, H-3′b), 3.81 (3H, *s*, 2-OCH_3_), 3.83 (3H, *s*, 3-OCH_3_), 3.74 (3H, *s*, 5-OCH_3_), 3.29 (2H, *d*, 6.5, H-1′). ^13^C-NMR (125 MHz, acetone-*d*_6_, *δ* ppm): 151.5 (C-2), 148.6 (C-4), 143.2 (C-5), 138.1 (C-2′), 119.6 (C-1), 115.4 (C-6), 114.3 (C-3′), 98.7 (C-3), 55.8, 55.7, 55.5 (4-OCH_3_, 2-OCH_3_, and 5-OCH_3_ signals could be interchanged), and 33.4 (C-1′). These data were consistent with those reported in the literature [29].

Coniferyl aldehyde (**PA4**). White amorphous solid. The ^1^H-NMR (400 MHz, methanol-*d*_4_) data were consistent with those reported in the literature [30].

Chrysophanol (**PA5**). Yellow amorphous solid. ^1^H-NMR (500 MHz, methanol-*d_4_*, *δ* ppm, *J* in Hertz): 12.05 (1H, *s*, 8-OH), 11.95 (1H, *s*, 1-OH), 7.83 (1H, *t*, 8.0, H-6), 7.79 (1H, *dd*, 8.0, 1.0, H-5), 7.63 (1H, *d*, 1.0, H-4), 7.36 (1H, *dd*, 8.0, 1.0, H-7), 7.20 (1H, *brs*, H-2), 2.50 (3H, *s*, H-11). ^13^C-NMR (125 MHz, methanol-*d_4_*, *δ* ppm): 163.3 (C-1), 124.9 (C-2), 150.7 (C-3), 121.7 (C-4), 115.0 (C-4a), 120.3 (C-5), 138.3 (C-6), 125.2 (C-7), 163.7 (C-8), 116.7 (C-8a), 190.0 (C-9), 114.7 (C-9a), 182.4 (C-10), 134.8 (C-10a), and 20.1 (C-11). These data were consistent with those reported in the literature [31].

Tribulusimide D (**PA6**). Colorless oil. ^1^H-NMR (500 MHz, acetone-*d*_6_, *δ* ppm, *J* in Hertz): 7.33 (*brs*; H-2), 6.87 (*d*, *J =* 8.0; H-5), 7.26 (*dd*, *J =* 6.5, 9.0; H-6), 7.59 (*d*, *J =* 16.0; H-7), 6.39 (*d*, *J =* 16.0; H-8), 7.57 (*d*, *J =* 6.5; H-2′), 7.14 (*d*, *J =* 8.0; H-5′), 7.47 (*brs*; H-6′), 3.92 (*s*; 3-OCH_3_), 3,72 (*s*; 3′-OCH_3_). ^13^C-NMR (125 MHz, acetone-*d*_6_, *δ* ppm): 171.4 (C-9), 168.8 (C-7′), 150.4 (C-4′), 149.7 (C-4), 147.9 (C-3), 147.8 (C-3′), 145.7 (C-7), 126.6 (C-1), 125.5 (C-6′), 124.9 (C-6), 123.9 (C-5), 122.6 (C-1′), 119.9 (C-5′), 116.1 (C-8), 115.6 (C-2′), 111.4 (C-2), 56.4 (3-OCH_3_), and 51.5 (3′-OCH_3_). The NMR data were consistent with those reported previously [32].

5-Hydroxymethylfurfural (**PA7**). Colorless oil. ^1^H-NMR (500 MHz, acetone-*d*_6_) and ^13^C-NMR (125 MHz, acetone-*d*_6_). These data were consistent with those reported in the literature [33].

### 2.3. Alpha-Glucosidase Inhibition of Extracts, Fractions, and Compounds from D. linearis and P. adenophylla

The results of alpha-glucosidase inhibition for both extracts and fractions obtained from *D. linearis* and *P. adenophylla* are presented in Table 1. Additionally, the evaluation of the isolated compounds for their alpha-glucosidase inhibitory activity is presented in Table 2. From *D. linearis*, compounds **DL3**, **DL5**-**DL8**, and **DL10**-**13** exhibited good inhibition with IC_50_ values ranging from 67.1 to 282.1 µM, whereas others were inactive. From *P. adenophylla*, compounds **DL10** and **PA4**-**PA6** showed potent inhibition with IC_50_ values of 67.1, 249.7, 98.2, and 76.2 µM, respectively.

**Figure 4:**
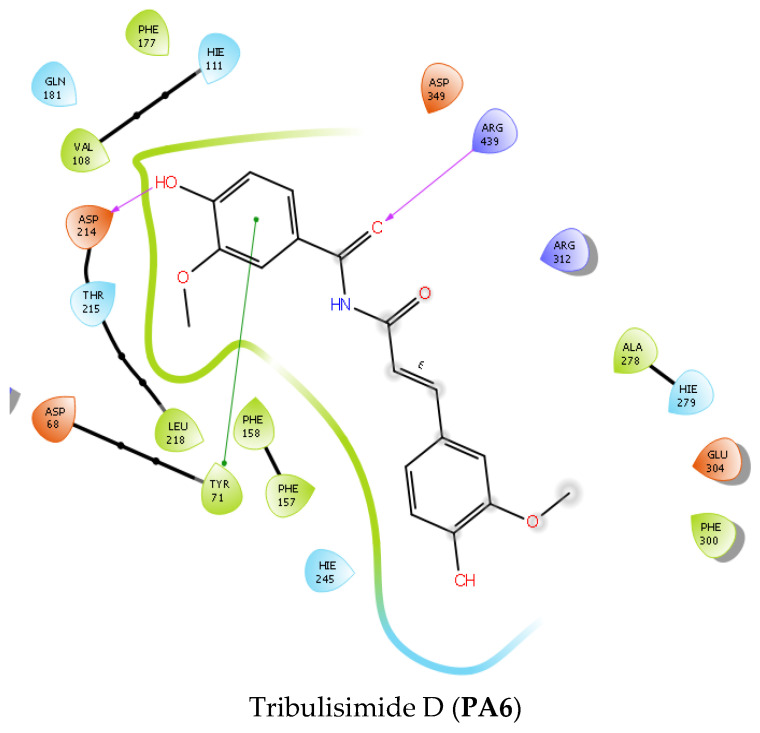

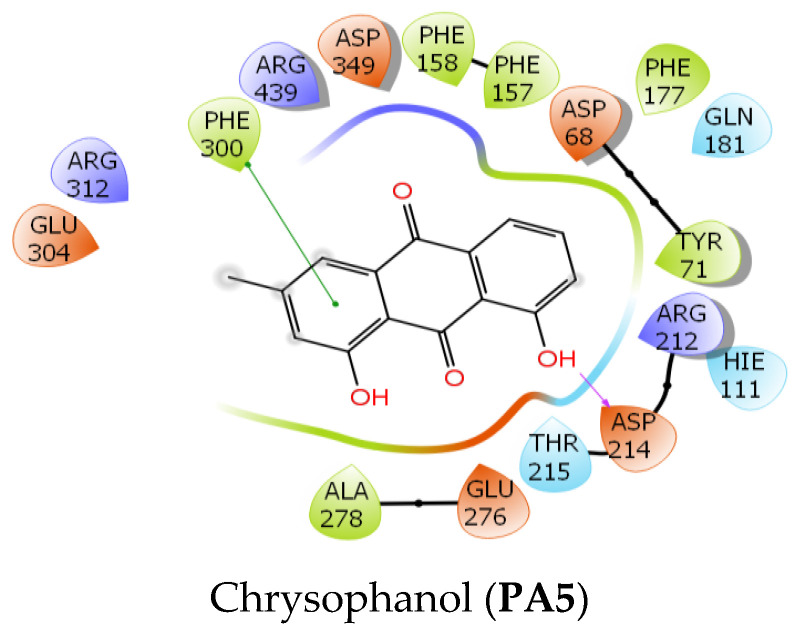
The 2D interactions of two ligand–protein complexes: **PA5** and **PA6**.

**Figure 5:**
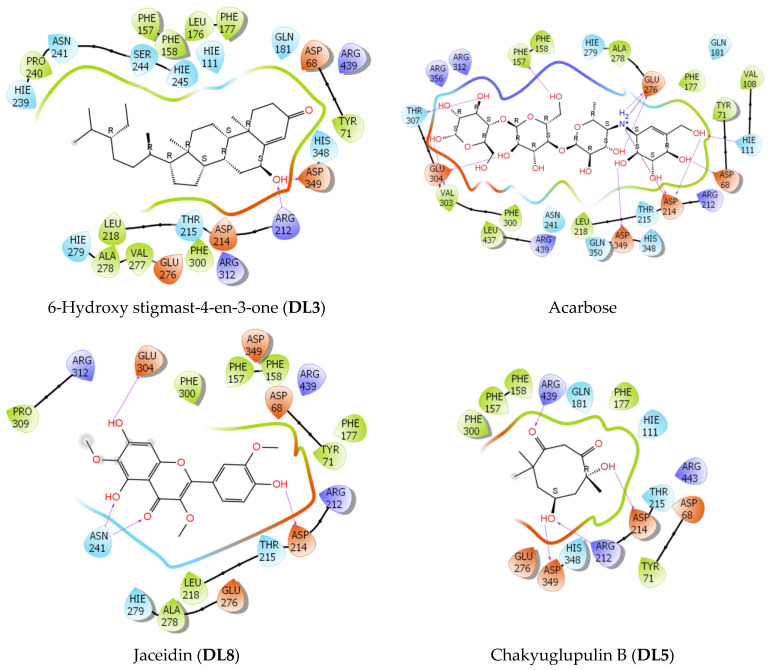
The 2D interactions of three ligand–protein complexes (**DL3**, **DL5**, and **DL8**) in comparison with acarbose.

The molecular docking models of **DL1** [34], **DL2** [35], **DL6** [36], **DL10** [37], **DL11** [38], **DL12** [39], **DL13** [39], and **PA4** [40] were investigated extensively, so no in silico analysis of those compounds were performed in the current study. Molecular docking studies were applied to compounds **DL3**, **DL5**, **DL8**, **PA5**, and **PA6**. The results are shown in Table 3 and Figure 4 and Figure 5.

## 3. Discussion

### 3.1. Chemical Composition of D. linearis and P. adenophylla

Until now, 21 compounds have been reported previously for *D. linearis* (see Figure S21). Most of them are glycosides with 1–3 sugar units, and they appear in polar fractions. From the Vietnamese *D. linearis*, three steroids (**DL1**–**DL3**), eight flavonoids (**DL6**–**DL13**), one diphenylketone (**DL4**), and an 8-member-ring compound (**DL5**) were isolated. In 2021, Zakaria and co-workers undertook multiple analytic methods to determine phenolic compounds and triterpenes from the Malaysian *Dicranopteris linearis* leaves without isolation [41]. To the best of our knowledge, compounds **DL1** and **DL3**–**DL10** were reported for the first time in the genus *Dicranopteris*.

Only six compounds were reported previously for *P. adenophylla* (see Figure S22). All of them are common compounds found in many plants. Using bioactive-guided isolation on the Vietnamese *P. adenophylla*, eight compounds were successfully isolated. To the best of our knowledge, compounds **PA1**–**PA6** were reported for the first time in the genus *Psychotria*. Many previous phytochemical investigations on the *Psychotria* plants focused on alkaloids using an alkaloid-isolation procedure [42,43,44,45]. Interestingly, the alkaloid tribulusimide D (**PA6**) was detected in *P. adenophylla*, representing a new alkaloid type within the genus *Psychotria*.

### 3.2. Alpha-Glucosidase Inhibition of Extracts, Fractions, and Compounds from D. linearis and P. adenophylla

Our literature review showed that crude extracts of both *D. linearis* and *P. adenophylla* have not been evaluated for alpha-glucosidase inhibition. Very little is known about the alpha-glucosidase inhibitory activity of the *Dicranopteris* and *Psychotria* plants. Recent studies regarding the alpha-glucosidase inhibition of the crude extracts from other *Dicranopteris* and *Psychotria* plants, *D. caudata*, *P. malayana*, and *P viridiflora*, were performed, indicating the potent inhibition of the extracts of these plants [46,47,48].

Although the crude methanol extract of *D. linearis* showed good activity, its derived extracts and fractions showed weaker alpha-glucosidase inhibition (Table 1). This indicated that the combination of all components of *D. linearis* might increase the activity. Further antidiabetic investigation of this plant should be conducted on the crude extract.

As seen in Table 1, the ethyl acetate extract of *D. linearis* had an IC_50_ value of 124.1 µg/mL. Therefore, it was further fractionated to obtain five fractions (EA1–EA5). Fractions EA2–EA4 were chosen for further isolation based on their good alpha-glucosidase inhibition. Compounds **DL7** and **DL10**–**DL13** were isolated from these fractions. Two isoflavones, **DL6** and **DL7**, were isolated from the extract HEA. Those mentioned flavonoids are well-known alpha-glucosidase inhibitors that have been comprehensively studied. Particularly, the consistency between in vitro alpha-glucosidase inhibitory activity and in vivo data of luteolin (**DL10**) and daizein (**DL6**) was confirmed by various reports [37,49,50]. Quercetin (**DL11**), isoquercitin (**DL12**), and kaempferol (**DL13**) were potent inhibitors, and the two formers were non-competitive types [39,51]. Two isoflavones, genistein (**DL5**) and daizein (**DL6**), also inhibited alpha-glucosidase, which is consistent with previously published reports [36,52]. These compounds are abundant in the ethyl acetate extract of *D. linearis*, indicating that they determine the activity of the extract **EA**. Multi-oxygenated flavonoids **DL8** and **DL9** are less potent than the above-mentioned flavonoids. Their low inhibition might be affected by the presence of the 3-oMe group, which was reported previously by Nguyen et al., 2023 [53].

The EA extract and its derived fractions of *P. adenophylla* showed potent activities, with IC_50_ values ranging from 1.7 to 26.6 µg/mL, much lower than that of the crude MeOH extract (Table 1). However, the isolation of bioactive components from these extracts and fractions is limited due to their high lignin content. The detection of the mixture of undefined lignins was determined using NMR and HPLC methods (Figure S20.1–20.3). Such macromolecules were known to be potent alpha-glucosidase inhibitors [54]. Only three compounds, **PA5**–**PA7**, were isolated from the most active fraction, EA5, but these compounds do not reflect the activity of the starting fraction. These compounds showed moderate activity with IC_50_ values in the range of 76.2–249.7 µM. Tribulusimide D (**PA6**) was previously found in *Euphorbia dracunculoides* [55] and *Tribuli fructus* [32], showing significant hepatoprotective activity with an EC_50_ value of 13.46 µM.

The docking results showed that 5/7 ligands were able to bind to residues within the binding site of alpha-glucosidase. The steric effect with bulky groups prevented complexation with proteins of 2,2′,4,4′-tetra-tert-butyl benzophenone and 6-hydroxy stigmast-4-en-3-one. In order of docking score, acarbose had the lowest value of −17.3 kcal/mol while the studied compounds ranged from −7.4 to −4.3 kcal/mol. In terms of energy estimates using MM-GBSA, acarbose showed the lowest value at −128.2 kcal/mol, followed by 6-hydroxy stigmast-4-en-3-one (**DL3**) at -65.4 kcal/mol, and tribulisimide D (**PA6**) and jaceidin (**DL8**) in the same range at −43 kcal/mol. Chakyuglupulin B (**DL4**) exhibited a higher value of −29.3 kcal/mol. Chrysophanol (**PA5**) had the highest energy estimate at −24 kcal/mol. These results show some deviation from the experimental values, but overall, the experimental values of all the studied compounds fall within the range of −5.5 to −4.5 kcal/mol. This indicates that these compounds exhibit moderate inhibition of *Saccharomyces cerevisiae* alpha-glucosidase.

In terms of interactions, acarbose stood out due to its extensive occupancy of the chemical space and the significant presence of hydrogen bonding within the interaction networks. The inhibitory mechanism of acarbose ignited with the H-bond between the hydroxyl group of acarbose and Asp 214, whereas Glu 276, the residue responsible for catalyzing the hydrolysis of the normal 1,4-alpha-gluco bond, was engaged with two hydrogen bonds, one from acarbose’s nitrogen and one from the hydroxyl group. In addition, a salt bridge was also formed between the cationic ammonium and the carboxylate group of Glu 276. This observation was only visible when the nitrogen of acarbose was protonated in a physiological environment (pH = 7 ± 2), which was achieved using Ligprep. Therefore, Glu 276 could not hydrolyze the C-N bond between the glucose molecules of acarbose. Additionally, Asp 349, a residue considered a transition state stabilizer of diose or triose degradation, could also form a hydrogen bond with acarbose. According to the described mechanism, Figure 4 and Figure 5 and Table 3 indicate that among the three key residues (Asp 214, Glu 276, and Asp 349), all the studied compounds formed hydrogen bonds with either Asp 214 or both Asp 214 and Glu 276, except for compound B, which only formed a H-bond with the key residue Asp 349. This provides compelling evidence of their potential inhibitory effect on the enzyme’s activity, potentially impeding polysaccharide hydrolysis by *Saccharomyces cerevisiae* alpha-glucosidase.

The moderate activity of these ligands may be attributed to the specific structure of each substance, which prevents them from optimally filling the chemical space within the alpha-glucosidase binding site. As a result, there are easily accessible solvent regions deep inside the binding site, which reduces their inhibitory activity.

## 4. Materials and Methods

### 4.1. Source of the Plant Material

*D. linearis* leaves were collected in Ba Ria-Vung Tau Province, Vietnam, from June to July 2022. The scientific name was identified as *Dicranopteris linearis* (Burm. F.) Underw. by Dr. Dang Van Son (deposited as No UE-P017).

The leaves of *P. adenophylla* were collected in Ba Ria-Vung Tau Province, Vietnam, from May to July 2022. The scientific name of the material was identified as *P. adenophylla* by Dr. Dang Van Son. A voucher specimen (No UE-P018) was deposited in the herbarium of the Department of Organic Chemistry, Faculty of Chemistry, Ho Chi Minh University of Education, Ho Chi Minh City, Vietnam.

### 4.2. Isolation of Compounds DL1-DL13 from D. linearis

**Scheme 1:**
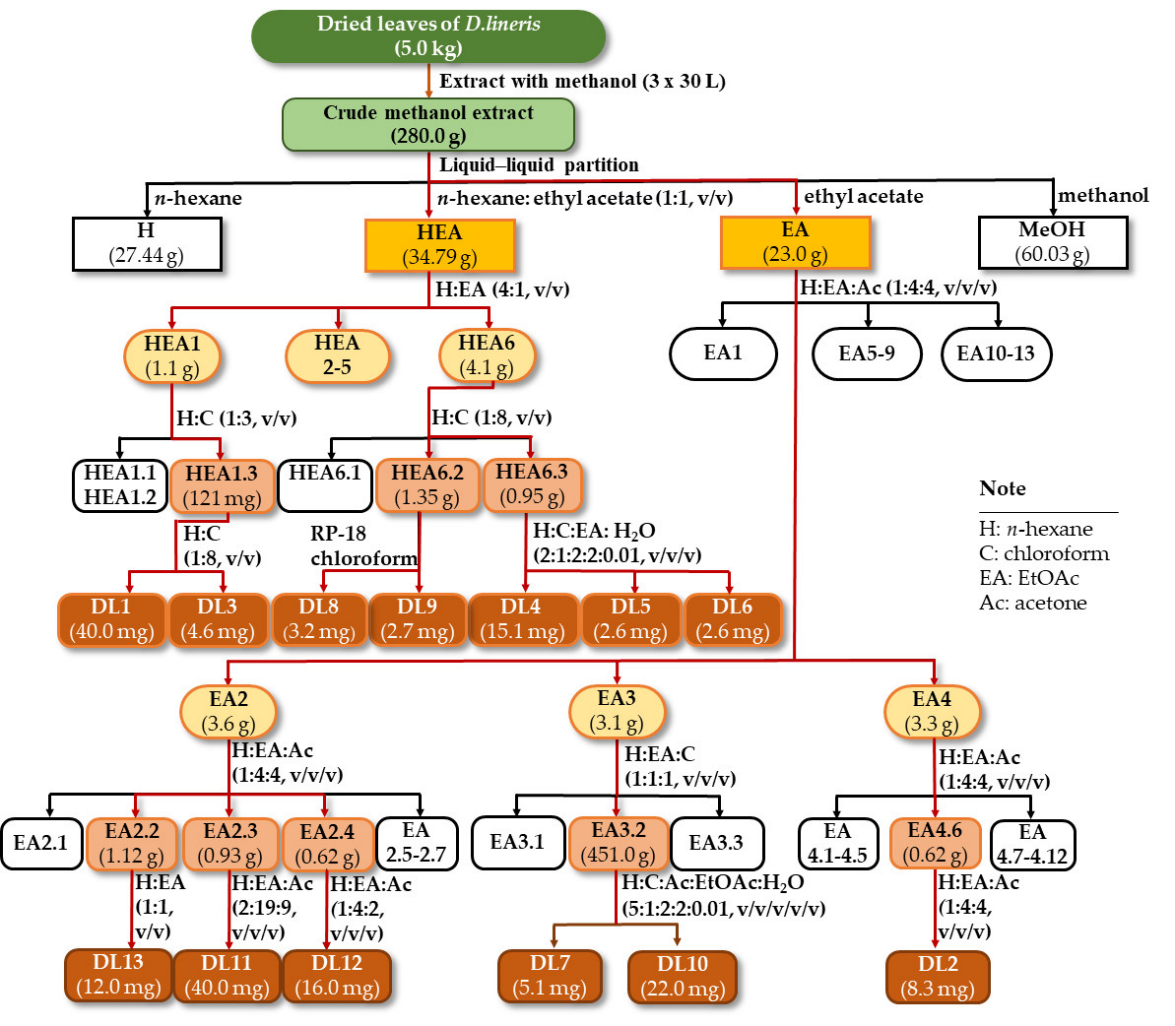
Isolation procedure of compounds **DL1**–**DL13** from *D. linearis.*

Dried and ground materials (5.0 kg) were exhaustedly extracted with methanol (3 × 30 L) at room temperature to provide a crude methanol extract (280.0 g). Liquid–liquid partition was applied to this extract using consecutive solvents *n*-hexane, *n*-hexane: EtOAc (1:1, *v*/*v*), EtOAc to give H (27.44 g), HEA (34.79 g), EA (23.00 g), and MeOH (60.03 g) extracts, respectively (Scheme 1).

The HEA extract (34.79 g) was subjected to a silica gel column chromatography (CC) using a mobile phase of *n*-hexane-EtOAc (4:1, *v*/*v*) to obtain six fractions (HEA1-HEA6). Fraction HEA1 (1.1 g) was applied to a silica gel CC using a gradient system of *n*-hexane: chloroform (1:3, *v*/*v*) to yield three subfractions (HEA1.1-HEA1.3). Subfraction HEA1.3 (121 mg) was subjected to a silica gel CC using a gradient system of *n*-hexane: chloroform (1:8, *v*/*v*) to afford compounds **DL1** (40.0 mg) and **DL3** (4.6 mg). Fraction HEA6 (4.1 g) was chromatographed on a silica gel CC and eluted with *n*-hexane: chloroform (1:9, *v*/*v*) to obtain three fractions (HEA6.1-HEA6.3). Fraction HEA6.2 (1.35 g) was rechromatographed on a silica gel CC and eluted with chloroform to afford compounds **DL8** (3.2 mg) and **DL9** (2.7 mg). Fraction HEA6.3 (0.95 g) was rechromatographed on a silica gel CC and eluted with *n*-hexane: chloroform: EtOAc: acetone: water (2:1:2:2:0.01, *v*/*v*/*v*/*v*/*v*) to yield compounds **DL4** (15.1 mg), **DL5** (2.6 mg), and **DL6** (2.6 mg).

Fractionation of the EA extract was performed on a silica gel CC and then eluted with *n*-hexane: EtOAc: acetone (1:4:4, *v*/*v*/*v*) to yield 13 fractions (EA1-EA13). Fraction EA2 (3.6 g) was subjected to a silica gel CC using a mobile phase as *n*-hexane: EtOAc: acetone (1:4:4, *v*/*v*/*v*) to obtain seven fractions (EA2.1-EA2.7). Fraction EA2.2 (1.12 g) was rechromatographed and then eluted with *n*-hexane: EtOAc (1:1, *v*/*v*) to yield compound **DL13** (12.0 mg). Fraction EA2.3 (0.93 g) was fractionated on a silica gel CC and eluted with *n*-hexane: EtOAc: acetone (2:19:9, *v*/*v*/*v*) to afford compound **DL11** (40.0 mg). Fraction EA2.4 (0.62 g) was purified on a silica gel CC and eluted with *n*-hexane: EtOAc: acetone (1:4:2, *v*/*v*/*v*) to obtain compound **DL12** (16.0 mg). Fraction EA3 (3.1 g) was applied to a silica gel CC using *n*-hexane: EtOAc: acetone (1:1:1, *v*/*v*/*v*) as a mobile phase to yield three fractions (EA3.1-EA3.3). Fraction EA3.2 (451 mg) was rechromatographed and then eluted with *n*-hexane: chloroform: acetone: EtOAc: water (5:1:2:2:0.01, *v*/*v*/*v*/*v*/*v*) to afford compounds **DL10** (22.0 mg) and **DL7** (5.1 mg). Fraction EA4 (3.3 g) was rechromatographed using a solvent system of *n*-hexane: EtOAc: acetone (1:4:4, *v*/*v*/*v*) to yield 12 fractions (EA4.1-EA4.12). Fraction EA4.6 (0.33 g) was chromatographed on a silica gel CC using *n*-hexane: EtOAc (1:3, *v*/*v*) as an eluent to yield compound **DL2** (8.3 mg).

### 4.3. Isolation of Compounds PA1-PA7 from P. adenophylla

**Scheme 2:**
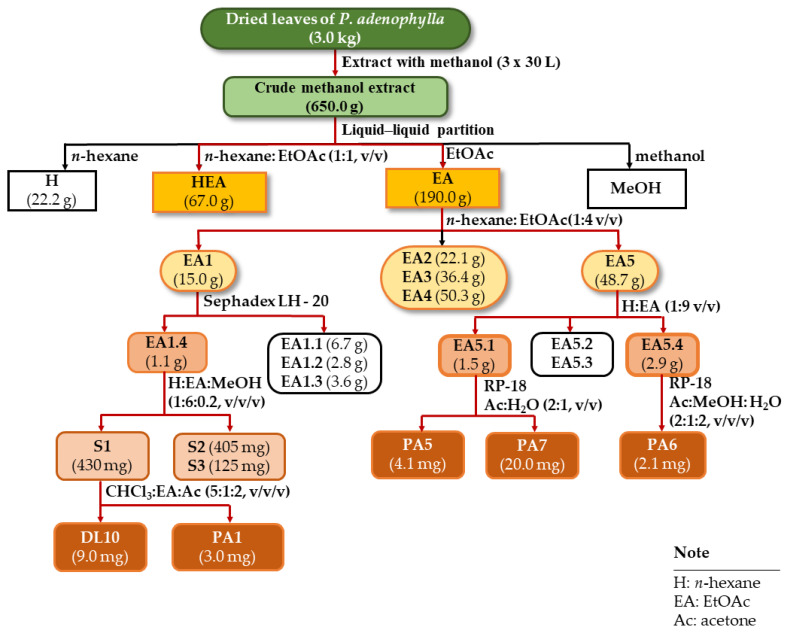
Isolation procedure of compounds **DL10**, **PA1**, and **PA5**-**7** from *P. adenophylla.*

Dried leaves of *P. adenophylla* (3.0 kg) were extracted using methanol (3 × 10 L) at room temperature. The filtrated solution was evaporated at reduced pressure to obtain a crude extract (650 g). This extract was separated into *n*-hexane extract (22.2 g, H), *n*-hexane: EtOAc extract (67 g, HEA), and EtOAc (190.0 g, EA) via liquid–liquid partition. The EA extract was applied to silica gel CC and eluted with *n*-hexane: EtOAc (1:4, *v*/*v*) to afford five fractions (EA1-EA5) (Scheme 2).

Fraction EA1 (15.0 g) was further subjected to a Sephadex LH-20 gel CC and eluted with MeOH to obtain four subfractions (EA1.1-EA1.4). Next, subfraction EA1.4 (1.1 g) was separated into three subfractions (EA1.4.1-EA1.4.3) using a silica gel CC and eluted with *n*-hexane: EtOAc: MeOH (1:6:0.2, *v*/*v*/*v*). Subfraction EA1.4.1 (430 mg) was purified using a silica gel CC with the solvent system of chloroform: EtOAc: acetone (5:1:2, *v*/*v*/*v*), resulting in the isolation of compounds **DL10** (9.0 mg) and **PA1** (3.0 mg). Fraction EA5 (48.7 g) was applied to a silica gel CC and eluted with *n*-hexane: EtOAc (1:9, *v*/*v*) to afford four subfractions (EA5.1-EA5.4). Subfraction EA5.1 (1.5 g) was continually chromatographed on a C-18 reverse-phase silica gel CC and eluted with acetone: water (2:1, *v*/*v*) to yield **PA5** (4.1 mg) and **PA7** (20 mg). Subfraction EA5.4 (2.9 g) was subjected to a C-18 reverse-phase silica gel CC and eluted with acetone: MeOH: water (2:1:2, *v*/*v*/*v*) to obtain compound **PA6** (2.1 mg).

The H extract was applied to silica gel CC and eluted with *n*-hexane: EtOAc: acetone (9:1:1, *v*/*v*/*v*) to afford 11 fractions (H1–H11). Fraction H4 (2.1 g) was further subjected to a silica gel CC and eluted with the solvent system of *n*-hexane: EtOAc: acetone (9:1:1, *v*/*v*/*v*) to yield seven subfractions (H4.1–H4.7). Next, subfraction H4.1 (123 mg) was separated into three fractions (S1–S3) on a silica gel CC and eluted with the same solvent system. Purifying fraction S1 (31 mg) on silica gel CC obtained compound **PA4** (3.5 mg). Subfraction H4.5 (67 mg) was subjected to a silica gel CC, using a solvent system of *n*-hexane: chloroform: MeOH: water (10:1:0.2:0.01, *v*/*v*/*v*/*v*) to afford three fractions (R1–R3). The same manner was applied to fraction R3 to afford a mixture of the compounds **PA2** and **PA3** (17.2 mg).

### 4.4. Alpha-Glucosidase Inhibition Assay

The enzyme alpha-glucosidase was derived from *Saccharomyces cerevisiae* (E.C 3.2.1.20) and compounds [acarbose, and 4-nitrophenyl *β*-D-glucopyranoside (*p*NPG)] were obtained from Sigma-Aldrich Co. (Saint Louis, MO, USA). The conditions followed that of our previous report with small modifications [56].

### 4.5. Molecular Docking Studies

The PDB structures of proteins (4j5t and 1t2p) were downloaded from the Protein Data Bank (PDB), while the 3D structures of ligands were modeled via the website chemicalize.com. After the conversion from PDB files into PDBQT format using AutodockTools, the docking study was designated on AutoDock4.2 using the Lamarckian genetic algorithm with 250 runs; the maximum number of evals was 25,000,000 (long) for each ligand–protein complex. The configurations with the most repetitions were employed to extract the estimated free energy as a scoring function for predicting the binding affinities to the macromolecular targets. Docking was carried out using the Maestro 12.5 software of Schrodinger Suites (Schrödinger Release 2020-3: Maestro, Schrödinger, LLC, New York, NY, USA, 2020). The protein of Saccharomyces cerevisiae alpha-glucosidase was obtained from the Uniprot database (P53341·MAL12_YEAST) and prepared using the Protein Preparation Wizard protocol. Next, seven reagents and an acarbose reference were generated and prepared using Ligprep [57]. As the protein itself does not contain ligands, the grid generation was based on Sitemaps detection’s suggestion, incorporating key residues such as Asp 214, Glu 276, and Asp 349 in the binding sites, with the highest site score of 1.1 [58]. In addition, the absence of a co-crystallized ligand in a binding site of the protein may not accurately represent the appropriate chemical space for ligand entry. Therefore, rigid docking can potentially lead to misinterpretation of ligand binding modes and inhibitory capacities. In contrast, Induced Fit docking (IFD) [59] allows the binding residues to dynamically adjust their positions to accommodate the ligands, allowing them to penetrate deeper into the binding sites. In the IFD process, specific restraints are implemented to prioritize the formation of h-bonds between the ligands and Asp 214, Glu 276, or Asp 349. After a glide docking SP (standard precision) with IFD, fine-tuning was performed using XP (extra precision)—IFD. The MM-GBSA method [60] was used to calculate the top two poses with the lowest docking scores for each ligand, and the result was chosen based on the best experimental fit. The experimental binding affinity was calculated based on the following equation:

$$\begin{array}{l} \Delta G_{\exp}=-\mathrm{RT}\ln IC_{50}\;(\mathrm{kcal}\cdot {\mathrm{mol}}^{-1})\\ \mathrm{wherein},\\ R=1.987\times 10^{-3}\;(\mathrm{kcal}\cdot K^{-1}\cdot \mathrm{mol})\\ T=300\;K \end{array}$$

${IC}_{50}$ is the concentration of a drug or inhibitor needed to inhibit a biological process or response by 50%.

### 4.6. Structure Elucidation of the Compounds

Gravity column chromatography was performed on silica gel 60 (0.040–0.063 mm, Merck, Darmstadt, Germany). Thin-layer chromatography (TLC) for checking the chromatographic patterns of fractions and isolated compounds was carried out on silica gel 60 F_254_ (Merck, Darmstadt, Germany) and the spots were visualized by spraying with 10% H_2_SO_4_ solution followed by heating. Specific rotations were obtained on a Jasco P-1010 polarimeter (Oklahoma City, OK, USA). The high-resolution electrospray mass spectra (HR-ESI-MS) were recorded using a MicrOTOF-Q mass spectrometer (Bruker, MA, USA). The Nuclear Magnetic Resonance (NMR) spectra were measured using a Bruker Avance 500 MHz spectrometer (Bruker, MA, USA).

### 4.7. HPLC Experiments Detected the Presence of Lignins in P. adenophylla

High-performance liquid chromatography (HPLC Agilent 1260 Infinity II) using the detector Diode Array detector (DAD) was employed for the analysis. A total of 35 µL of each sample (at the concentration of 1 mg/mL) was injected separately. A gradient system ofIN and water was used during the 60 min analysis: 5% to 10% ACN in 5 min, 10% to 30% ACN in 15 min, 30% to 80% ACN in 10 min, 80% to 100% ACN in 5 min, and 100% A in 5 min. A Luna C18 column (Phenomenex, 150 mm × 4.6 mm. i.d., 5 µm) and a C18 guard column (Phenomenex, Torrance, CA, USA) were employed for this analysis.

## 5. Conclusions

Twenty compounds were isolated from *D. linearis* and *P. adenophylla* plants using a bioactive-guided isolation procedure. Compounds **DL1, DL3**–**DL10**, and **PA1**–**PA6** were reported for the first time in the genera *Dicranopteris* and *Psychotria.* The isolated compounds were evaluated for alpha-glucosidase inhibition. Compounds **DL3**, **DL5**–**DL8**, **DL10**–**DL13**, and **PA4**–**PA6** showed potent inhibition with IC_50_ values ranging from 67.1 to 282.1 µM. A molecular docking study was applied to compounds **DL3**, **DL5**, **DL8**, **PA5**, and **PA6** for elucidating the mechanisms of alpha-glucosidase inhibition. Consequently, this study illustrated that *D. linearis* and *P. adenophylla* might be good potential natural sources to develop new antidiabetic preparations.

## Figures and Tables

**Table 1:** Alpha-glucosidase inhibition (IC_50_) by extracts and fractions.

Bio-Source	Extracts	IC_50_ µg/mL
***D. linearis***	Crude MeOH	31.1 ± 0.03
	*n*-Hexane (Extract **H**)	>300
	*n*-Hexane: ethyl acetate (Extract **HEA**)	195.0 ± 0.6
	Ethyl acetate (Extract **EA**)	124.1 ± 2.4
	Methanol (Extract **M**)	>300
	Fraction **HEA1**	>300
	Fraction **HEA2**	275.0 ± 4.6
	Fraction **HEA3**	>300
	Fraction **HEA4**	>300
	Fraction **HEA5**	>300
	Fraction **HEA6**	243.0 ± 2.9
	Fraction **EA1**	>300
	Fraction **EA2**	137.0 ± 2.9
	Fraction **EA3**	261.0 ± 8.7
	Fraction **EA4**	87.8 ± 1.1
	Fraction **EA5**	>300
***P. adenophylla***	Crude MeOH	109.9 ± 1.2
	*n*-Hexane (Extract **H**)	56.6 ± 3.5
	*n*-Hexane: ethyl acetate (Extract **HEA**)	16.6 ± 0.5
	Ethyl acetate (Extract **EA**)	6.7 ± 0.4
	Methanol (Extract **M**)	3.4 ± 0.1
	Fraction **EA1**	15.5 ± 0.5
	Fraction **EA2**	16.6 ± 0.5
	Fraction **EA3**	26.6 ± 1.2
	Fraction **EA4**	16.5 ± 0.4
	Fraction **EA5**	1.7 ± 0.04
**Acarbose (positive control)**		201.9 ± 2.2

**Table 2:** Alpha-glucosidase inhibition (IC_50_) of isolated compounds.

Bio-Source	Compounds	IC_50_ µM
***D. linearis***	**DL1**	>300
	**DL2**	>300
	**DL3**	177.3 ± 3.6
	**DL4**	>300
	**DL5**	82.2 ± 2.2
	**DL6**	66.9 ± 1.7
	**DL7**	267.5 ± 3.4
	**DL8**	282.1 ± 9.2
	**DL9**	>300
	**DL10**	67.1 ± 1.2
	**DL11**	117 ± 1.9
	**DL12**	99.8 ± 1.2
	**DL13**	230 ± 2.7
***P. adenophylla***	**PA1**	>300
	**PA2 + PA3**	>300
	**PA4**	249.7 ± 11.4
	**PA5**	98.2 ± 2.2
	**PA6**	76.2 ± 2.9
	**PA7**	>300
**Acarbose (positive control)**		313.2 ± 3.4

**Table 3:** The XP-IFD docking scores, the experimental/estimated binding affinities, and the number of H-bonds networks in the ligand–protein complex.

Compounds	XP-IFD Docking Scores (kcal/mol)	Binding Affinity (kcal/mol)		Number of H-Bonds	H-Bonds Network
		MM-GBSA	Exp		
Chakyuglupulin B (**DL5**)	−4.3	−29.3	−5.6	4	Asp 214, Arg 212, Asp 349, Arg 439
Jaceidin (**DL8**)	−6.8	−43.2	−4.9	4	Asp 214, Glu 304, Asn 241, Asn 241
Chrysophanol (**PA5**)	−5.2	−24.0	−5.5	2	Asp 214, Glu 276
Tribulisimide D (**PA6**)	−7.4	−43.6	−5.7	2	Asp 214, Arg 439
6-hydroxy stigmast-4-en-3-one (**DL3**)	−7.5	−65.4	−5.1	2	Asp 349, Arg 212
Acarbose (positive control)	−17.3	−128.2	−4.8	11	Asp 214, Glu 276, Asp 349, Asp 68, Hie 111, Phe 157, Glu 304, Thr 307

## Data Availability

Data is contained within the article and supplementary material.

## Supplementary Materials

The following supporting information can be downloaded at: https://www.mdpi.com/article/10.3390/ph16091253/s1. Figure S1. NMR spectra of DL1. Figure S2. NMR spectra of DL2. Figure S3. NMR spectra of DL3. Figure S4. NMR spectra of DL4. Figure S5. NMR spectra of DL5. Figure S6. NMR spectra of DL6. Figure S7. NMR spectra of DL7. Figure S8. NMR spectra of DL8. Figure S9. NMR spectra of DL9. Figure S10. NMR spectra of DL10. Figure S11. NMR spectra of DL11. Figure S12. NMR spectra of DL12. Figure S13. NMR spectra of DL13. Figure S14. NMR spectra of PA1. Figure S15. NMR spectra of PA2 and PA3. Figure S16. NMR spectra PA4. Figure S17. NMR spectra PA5. Figure S18. MS and NMR spectra of PA6. Figure S19. NMR spectra PA7. Figure S20. NMR spectra and HPLC chromatogram of the mixture of lignins. Figure S21. Chemical structures of compounds previously reported from *D. linearis*. Figure S22. Chemical structures of compounds previously reported from *P. adenophylla*.
