# Supplementary material for: Bioactive-Guided Phytochemical Investigations, In Vitro and In Silico Alpha-Glucosidase Inhibition of Two Vietnamese Medicinal Plants Dicranopteris linearis and Psychotria adenophylla
Source: Pharmaceuticals (Basel). 2023 Sep 5;16(9):1253. doi: 10.3390/ph16091253 (PMC10538207; doi:10.3390/ph16091253)
Supplement: Supplementary file 1 [file pharmaceuticals-16-01253-s001.zip › pharmaceuticals-2574291-supplementary.pdf]

Supporting information

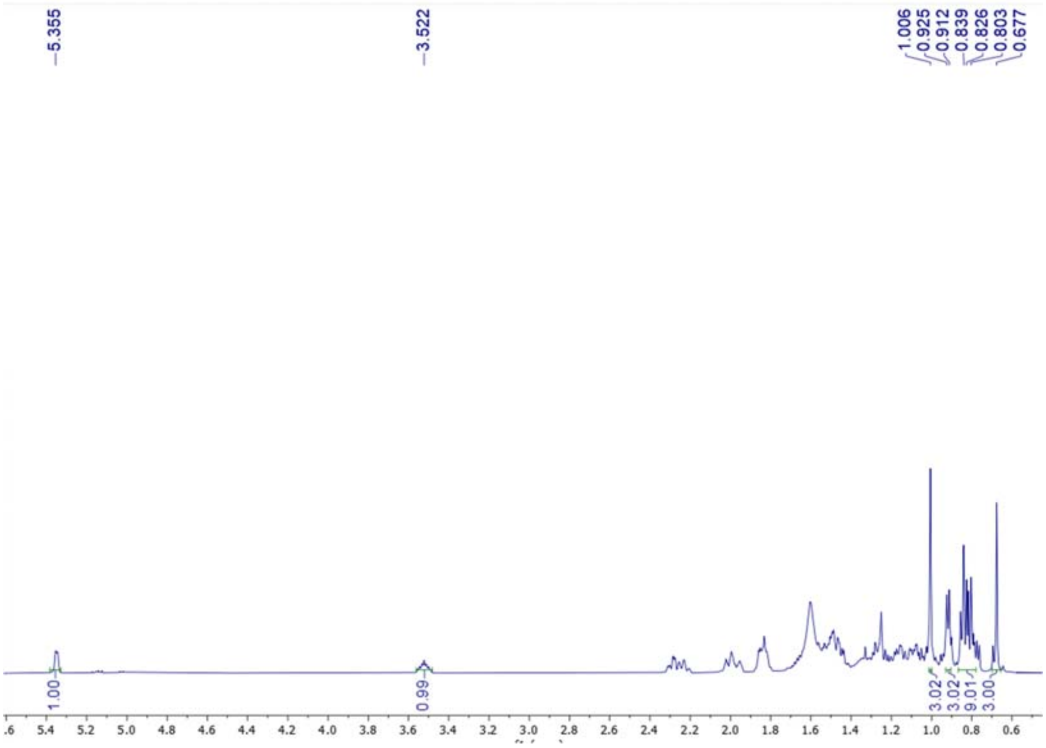

Figure S1.1. ^{1}H NMR spectrum of **DL1** (Chloroform–*d*)

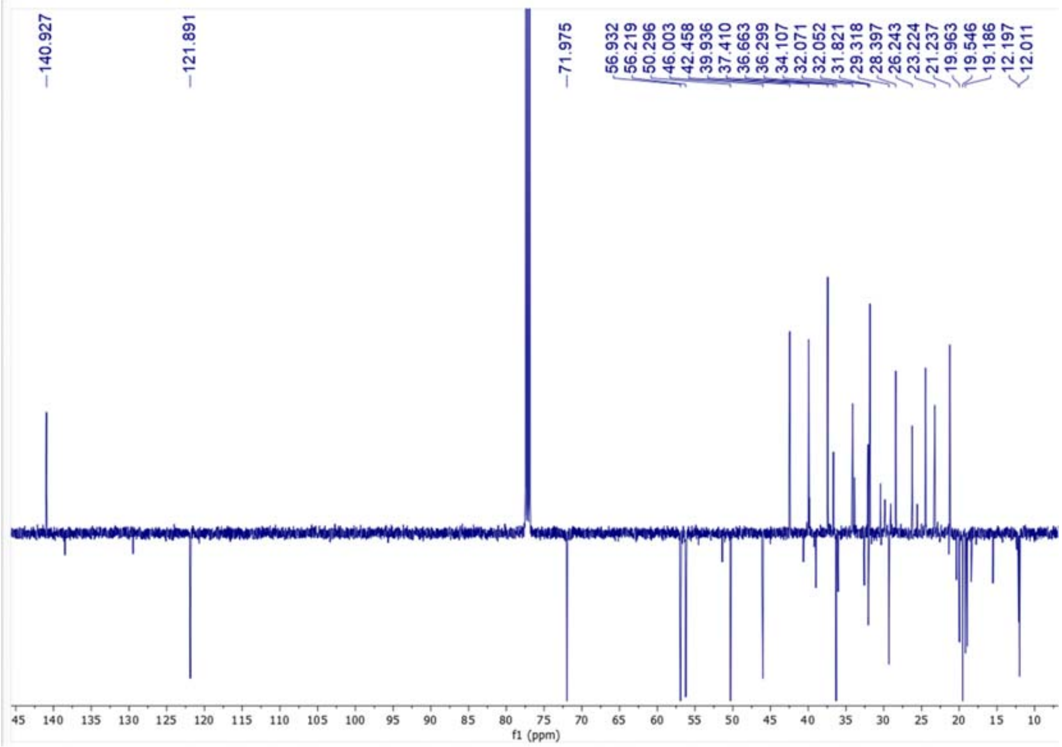

Figure S1.2. ^{13}C NMR spectrum of **DL1** (Chloroform–*d*)

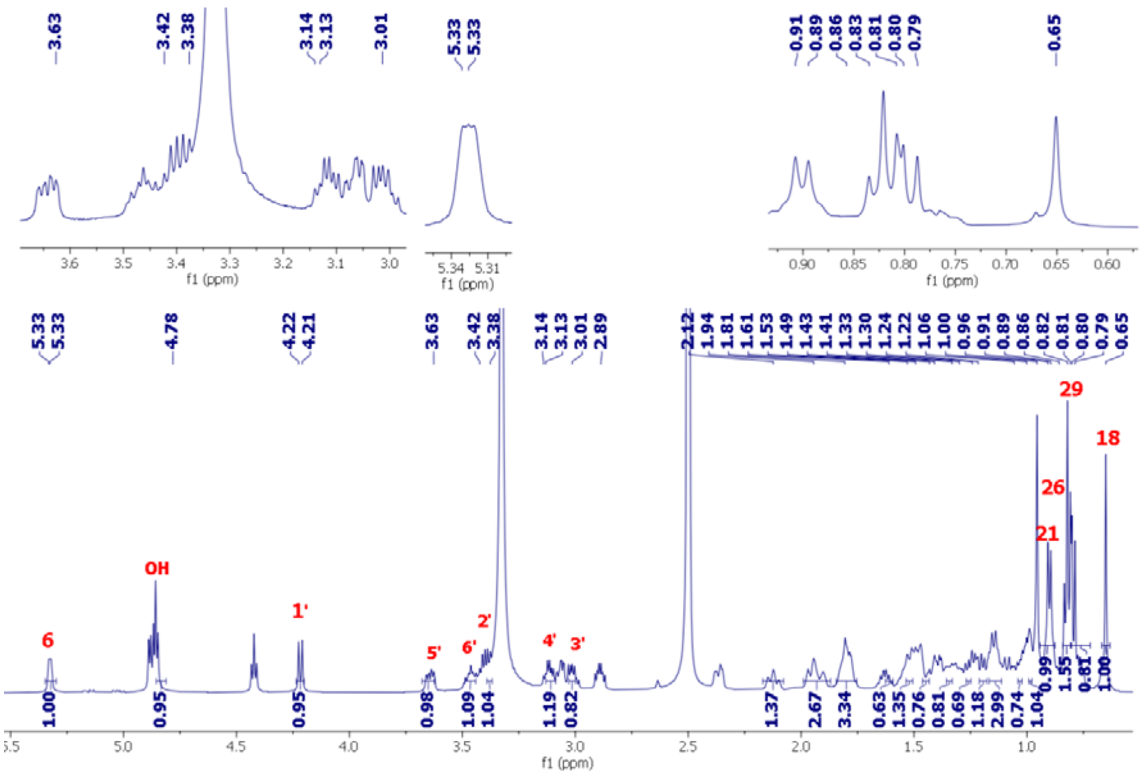

Figure S2.1. ^{1}H NMR spectrum of **DL2** (DMSO–d_6)

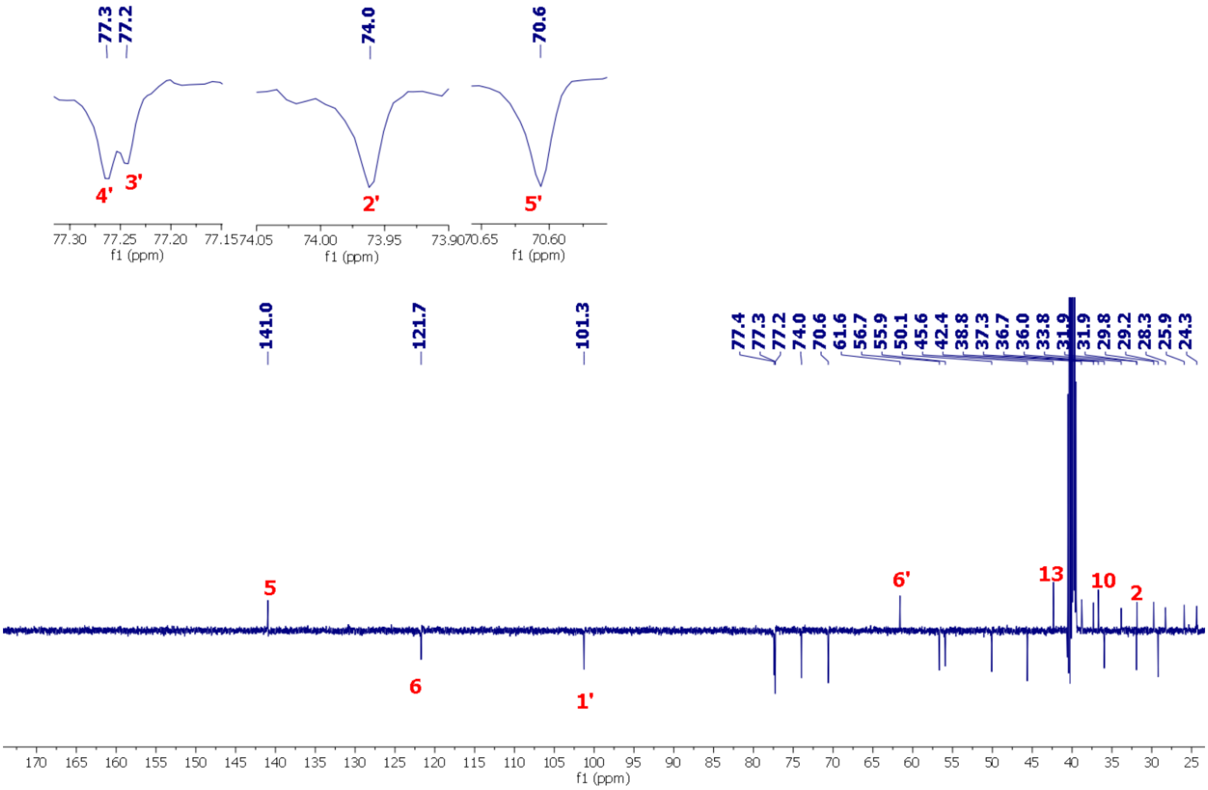

Figure S2.2. ^{13}C NMR spectrum of **DL2** (DMSO–d_6)

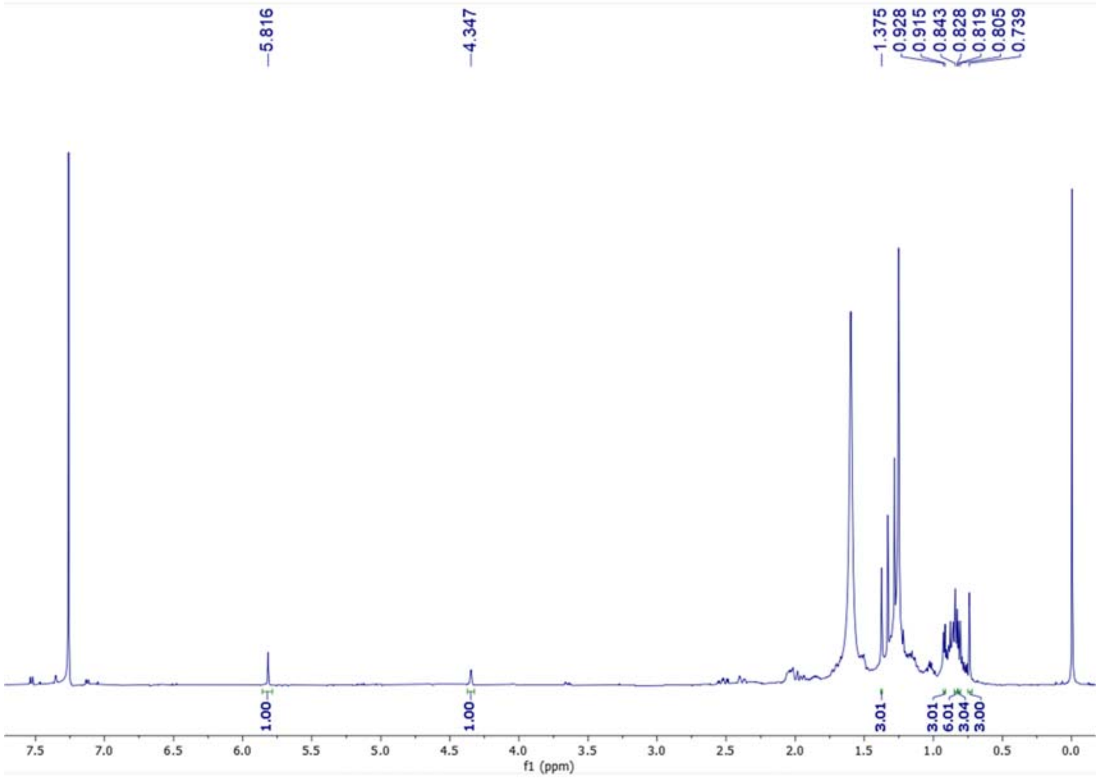

Figure S3.1. ^{1}H NMR spectrum of **DL3** (Chloroform–*d*)

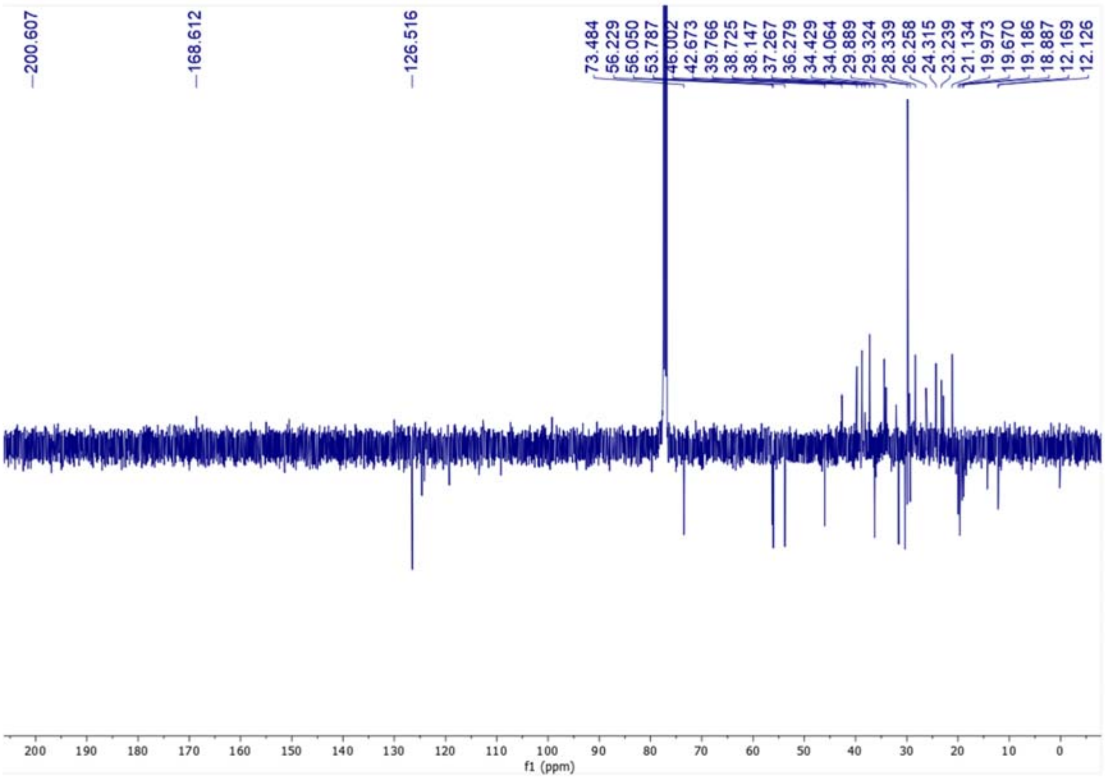

Figure S3.2. ^{13}C NMR spectrum of **DL3** (Chloroform–*d*)

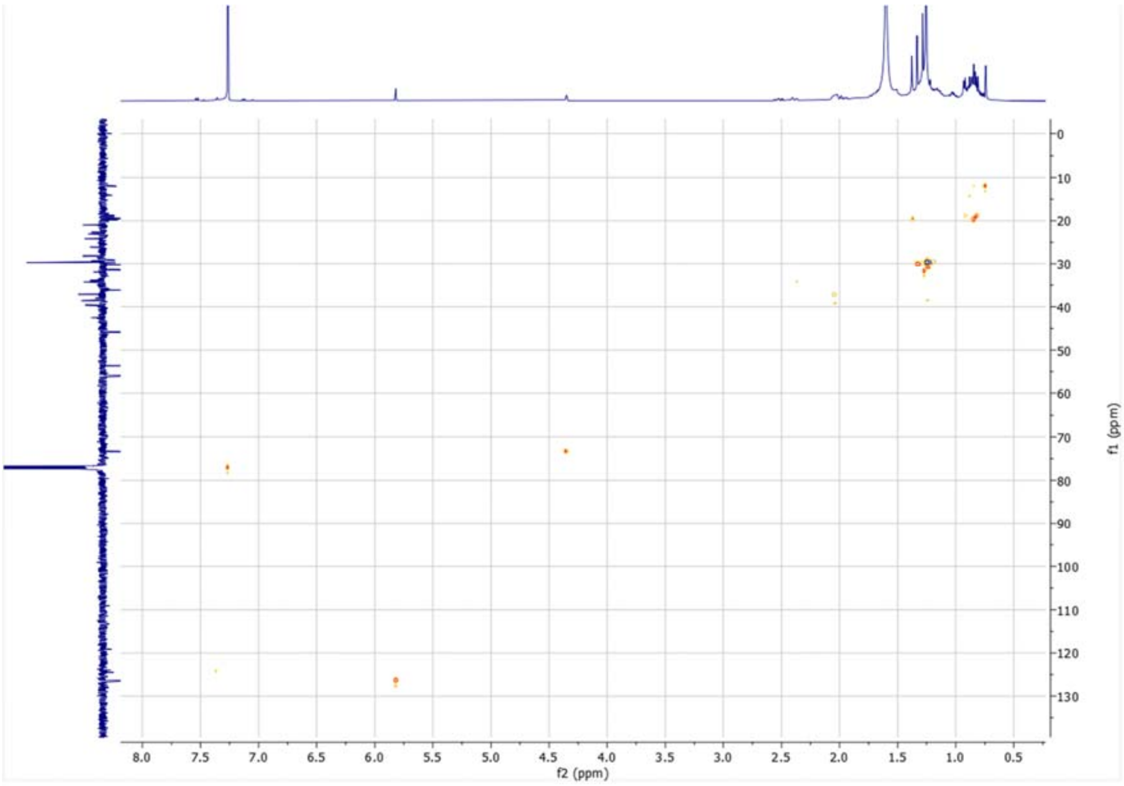

Figure S3.3. HSQC spectrum of **DL3** (Chloroform–*d*)

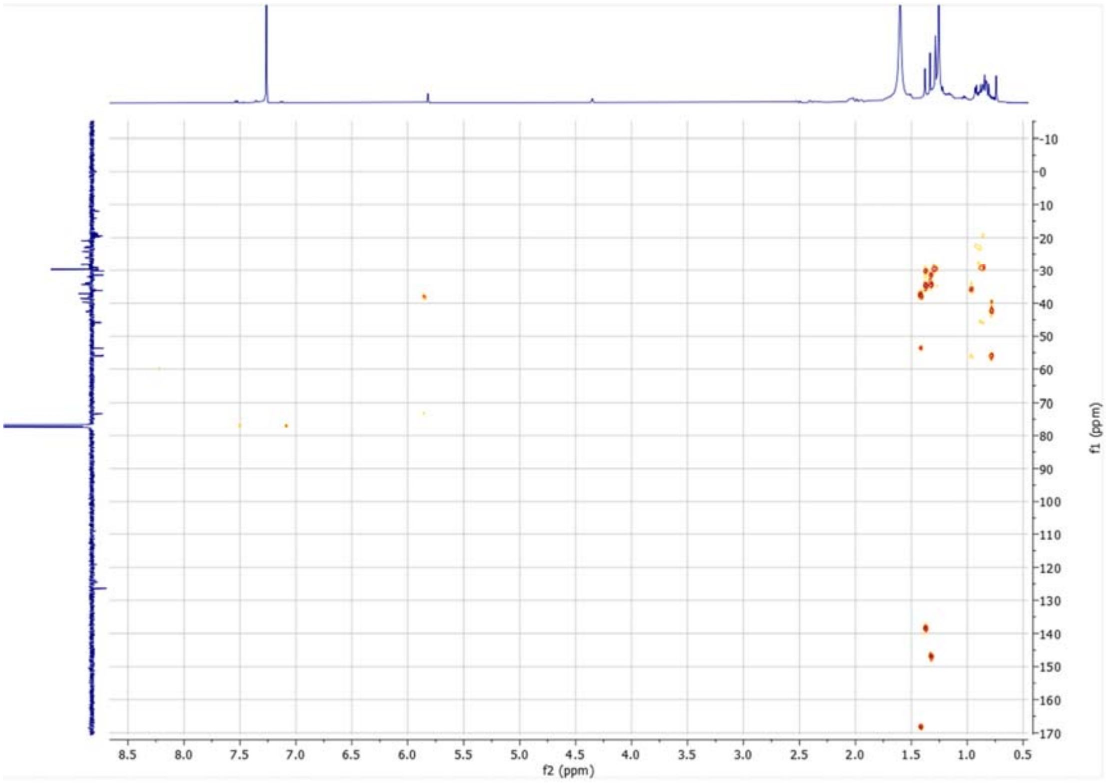

Figure S3.4. HMBC spectrum of **DL3** (Chloroform–*d*)

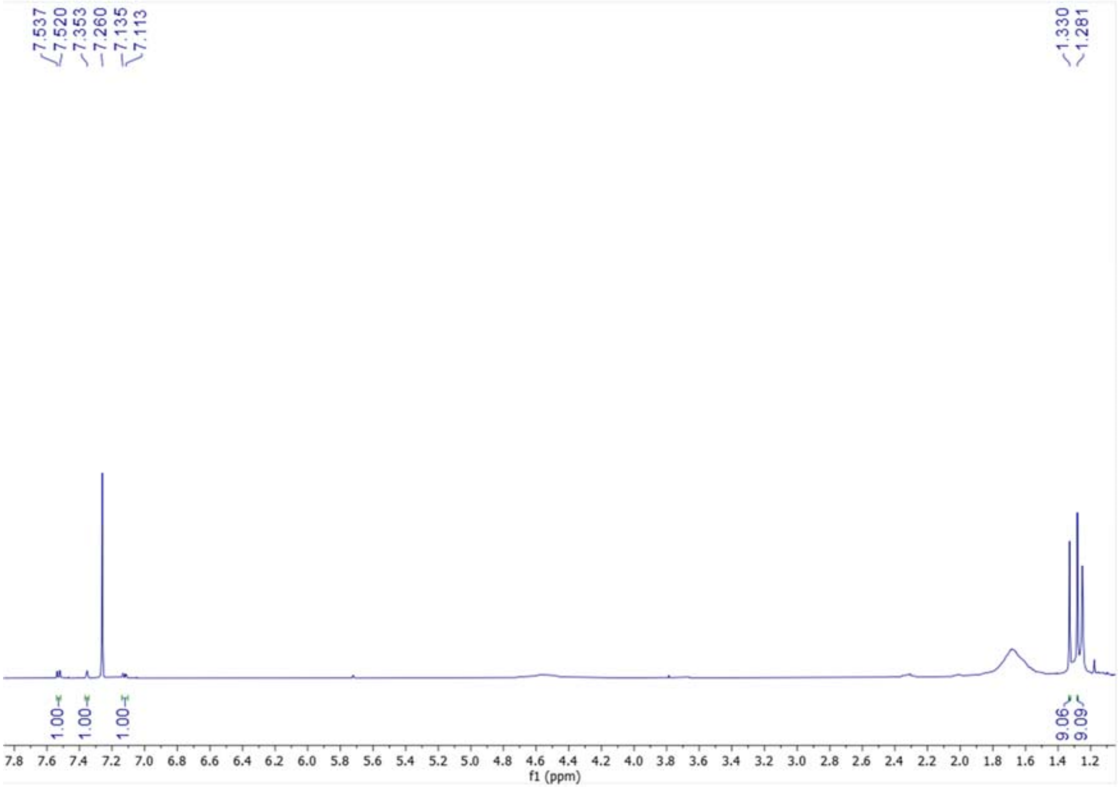

Figure S4.1. ^{1}H NMR spectrum of **DL4** (Chloroform–*d*)

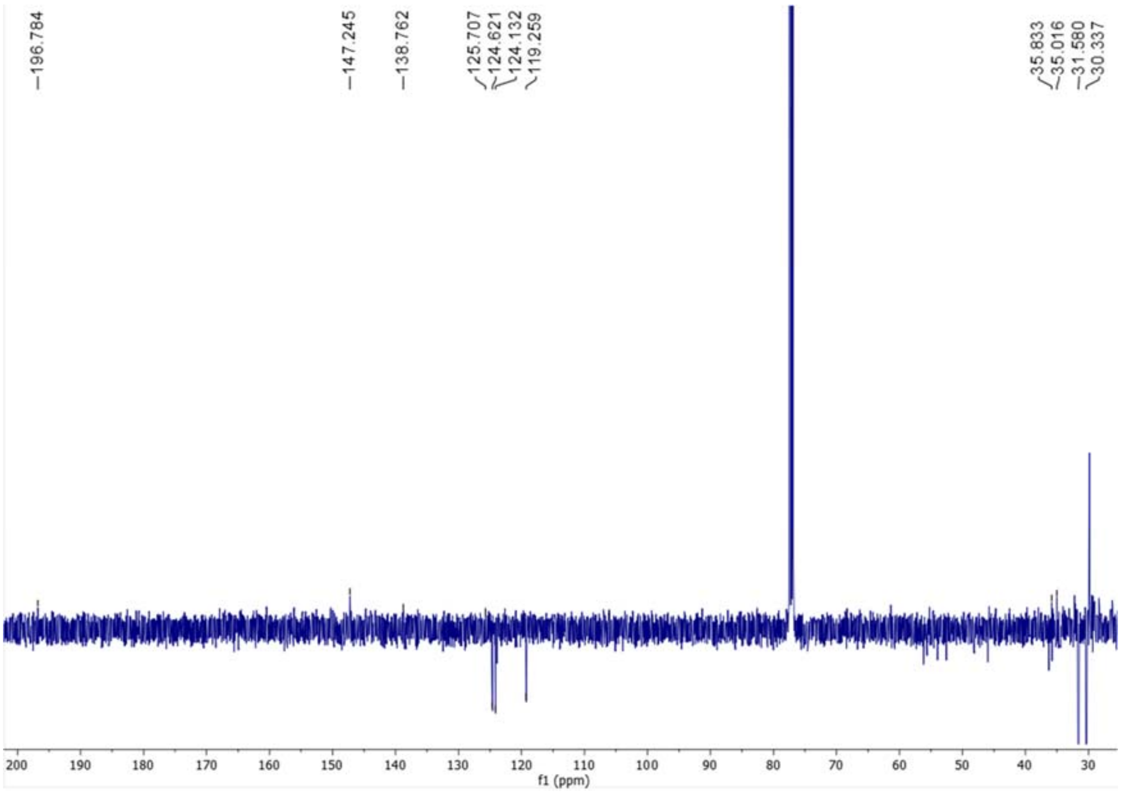

Figure S4.2. ^{13}C NMR spectrum of **DL4** (Chloroform–*d*)

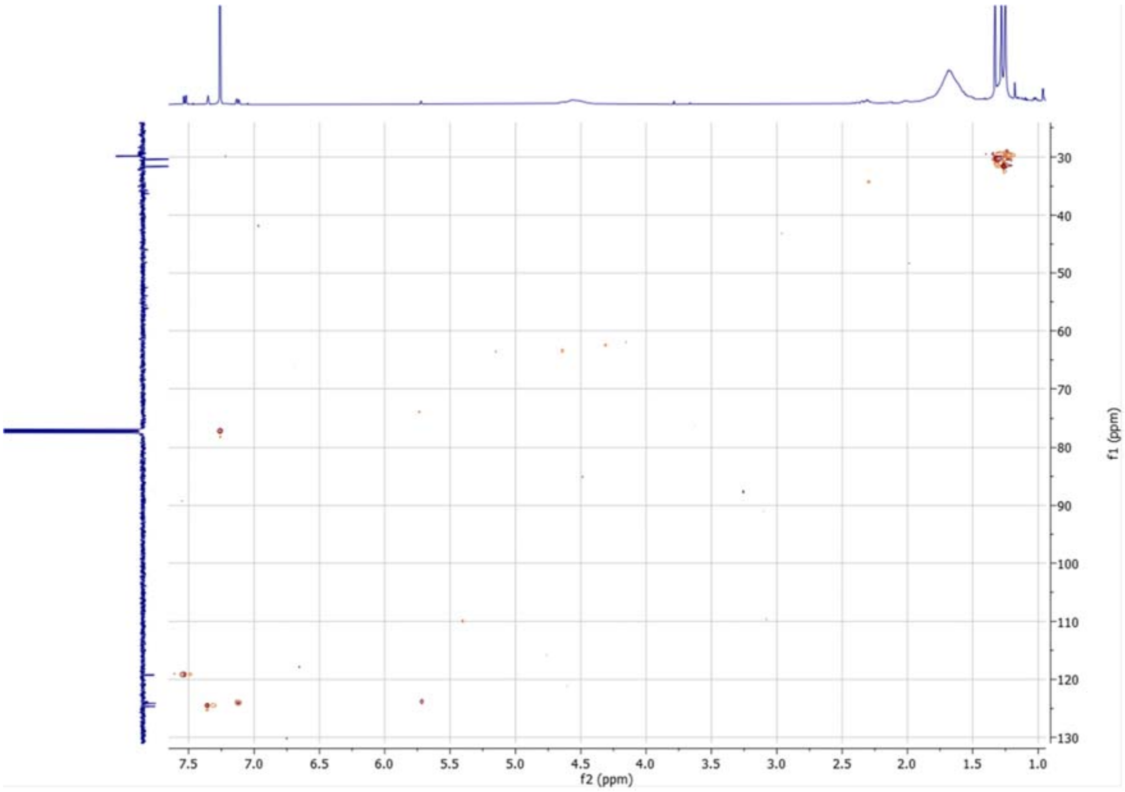

Figure S4.3. HSQC spectrum of **DL4** (Chloroform–*d*)

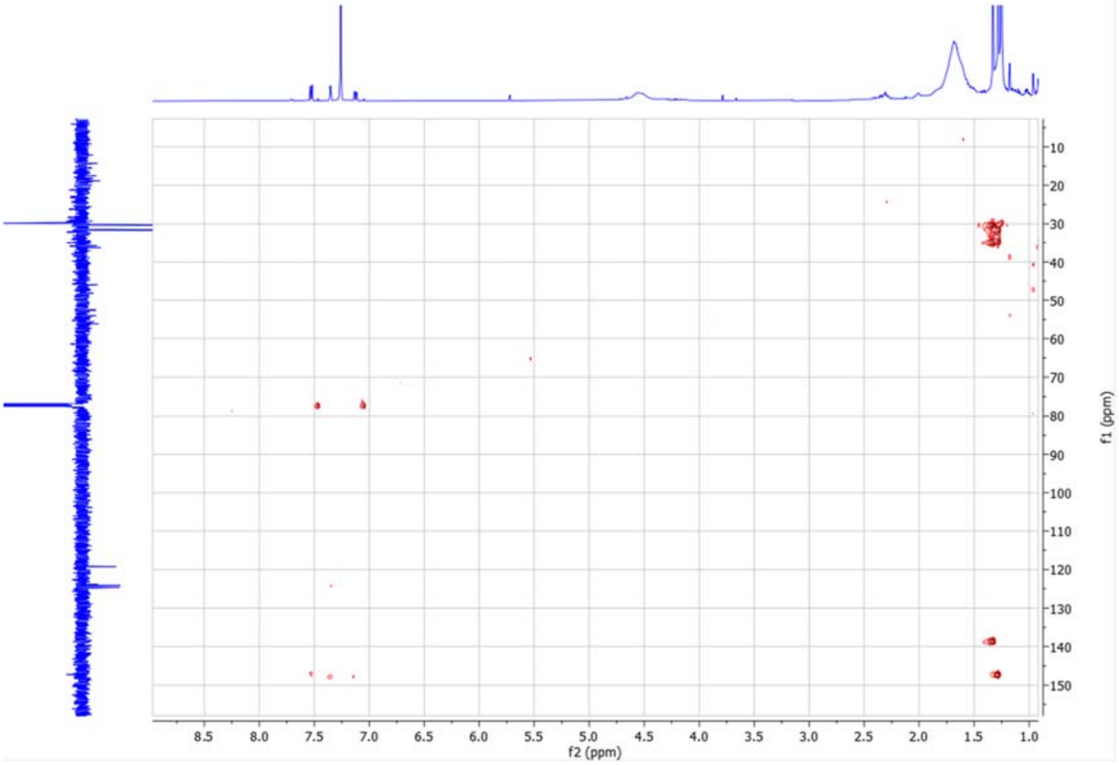

Figure S4.4. HMBC spectrum of **DL4** (Chloroform–*d*)

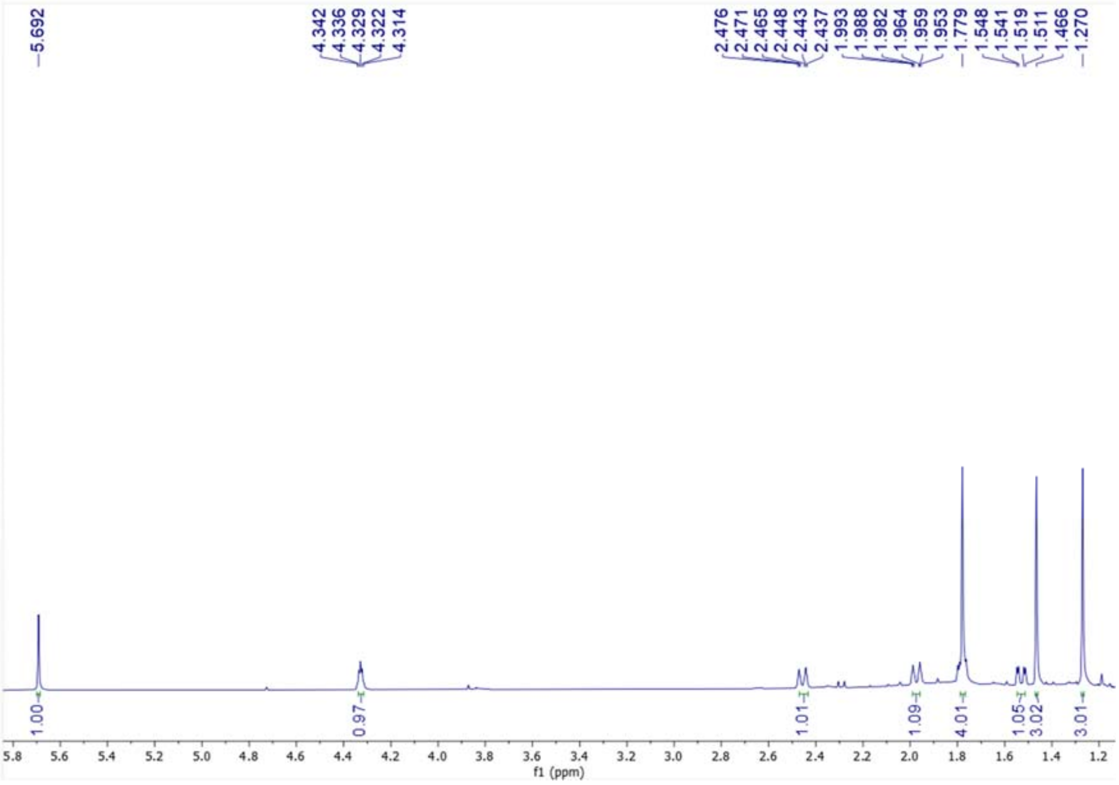

Figure S5.1. ^{1}H NMR spectrum of **DL5** (Chloroform–*d*)

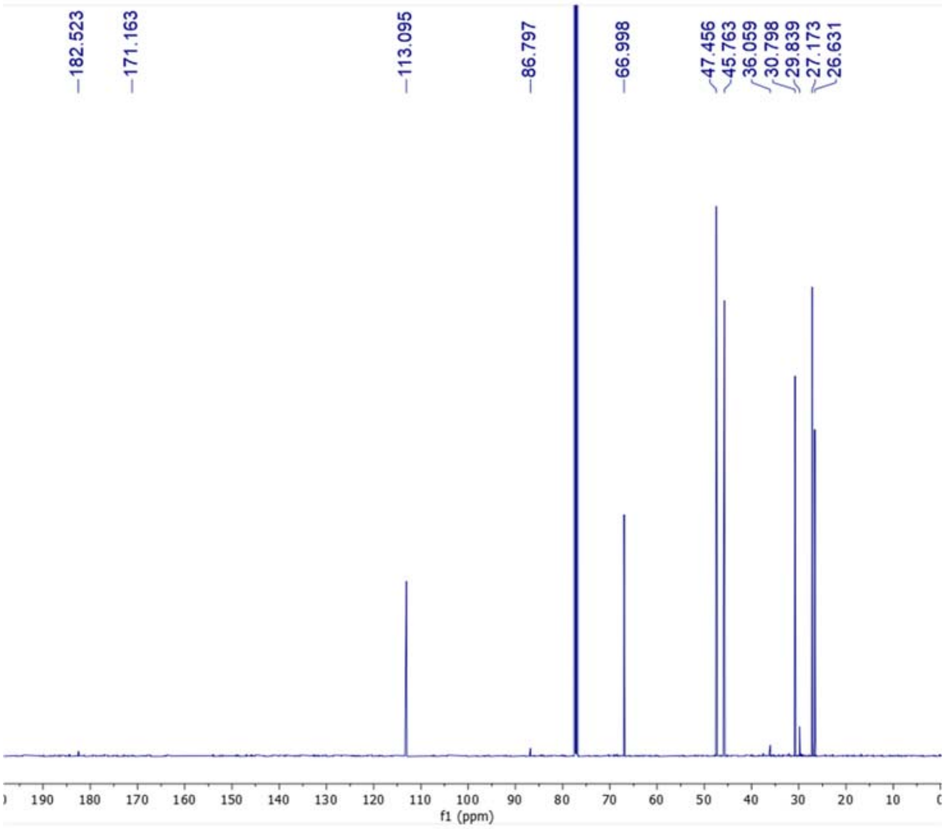

Figure S5.2. ^{13}C NMR spectrum of **DL5** (Chloroform–*d*)

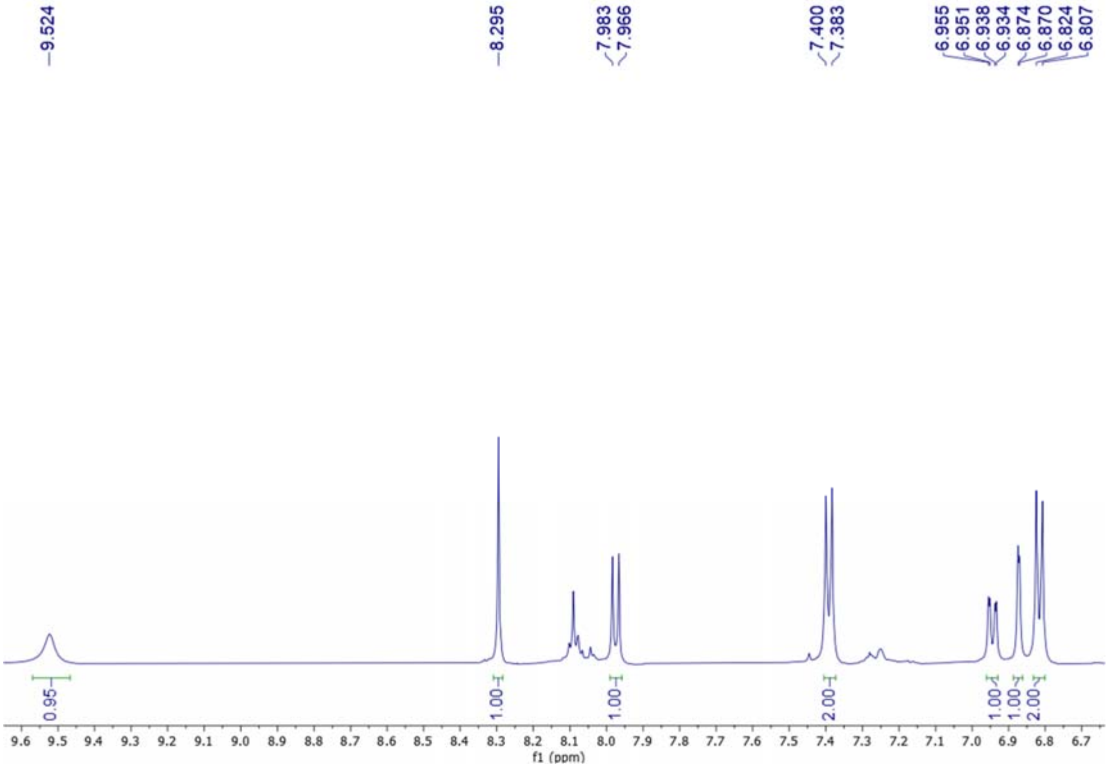

Figure S6.1. ^{1}H NMR spectrum of **DL6** (DMSO–d_6)

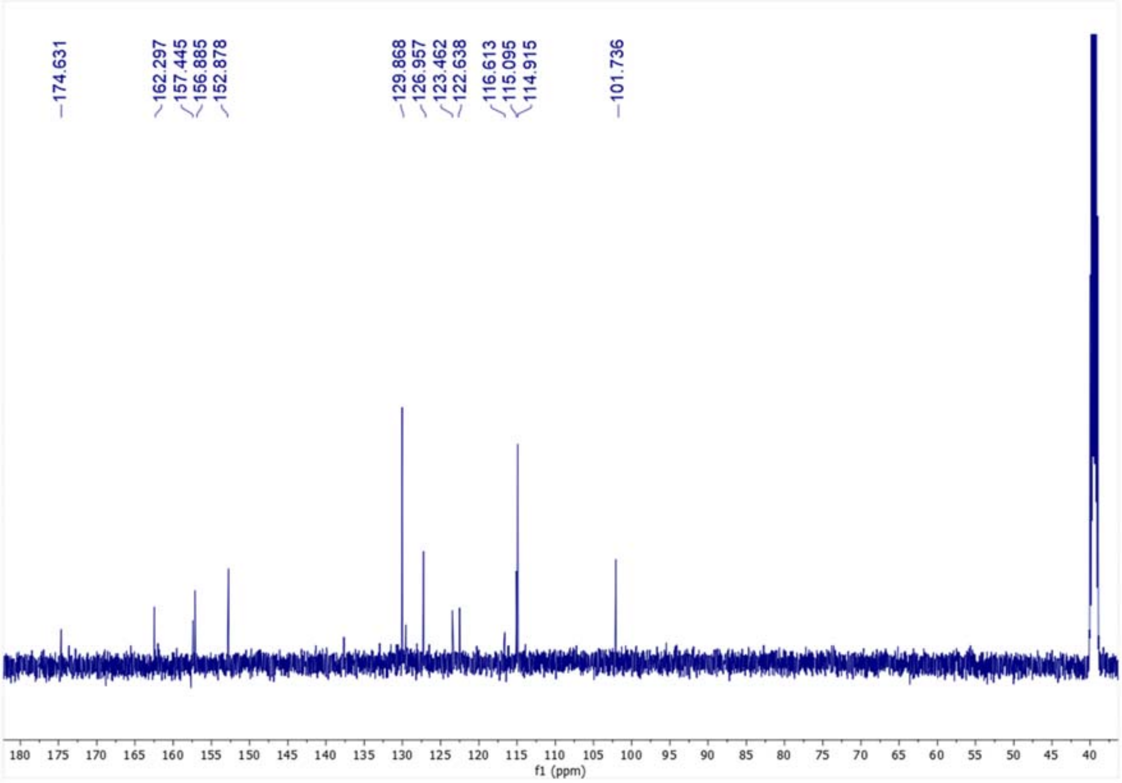

Figure S6.2. ^{13}C NMR spectrum of **DL6** (DMSO–d_6)

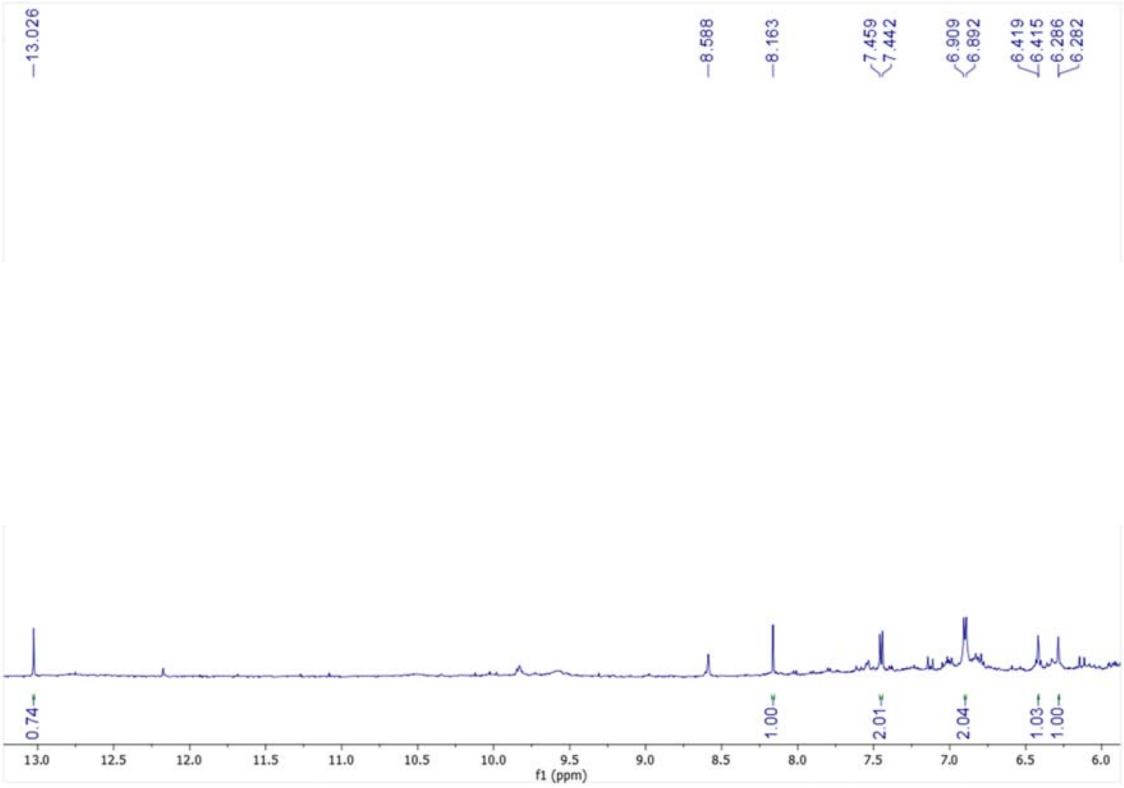

Figure S7.1. ^{1}H NMR and HMBC spectrum of **DL7** (Acetone–d_6)

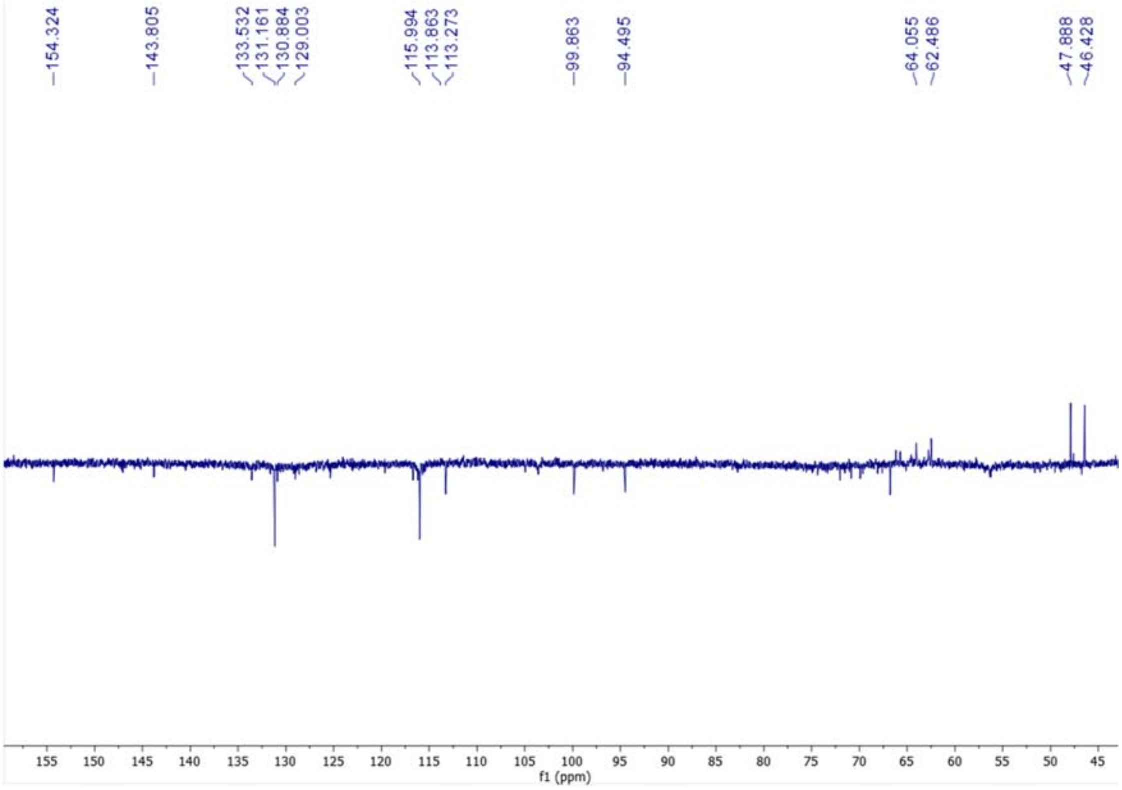

Figure S7.2. ^{13}C NMR and HMBC spectrum of **DL7** (Acetone–d_6)

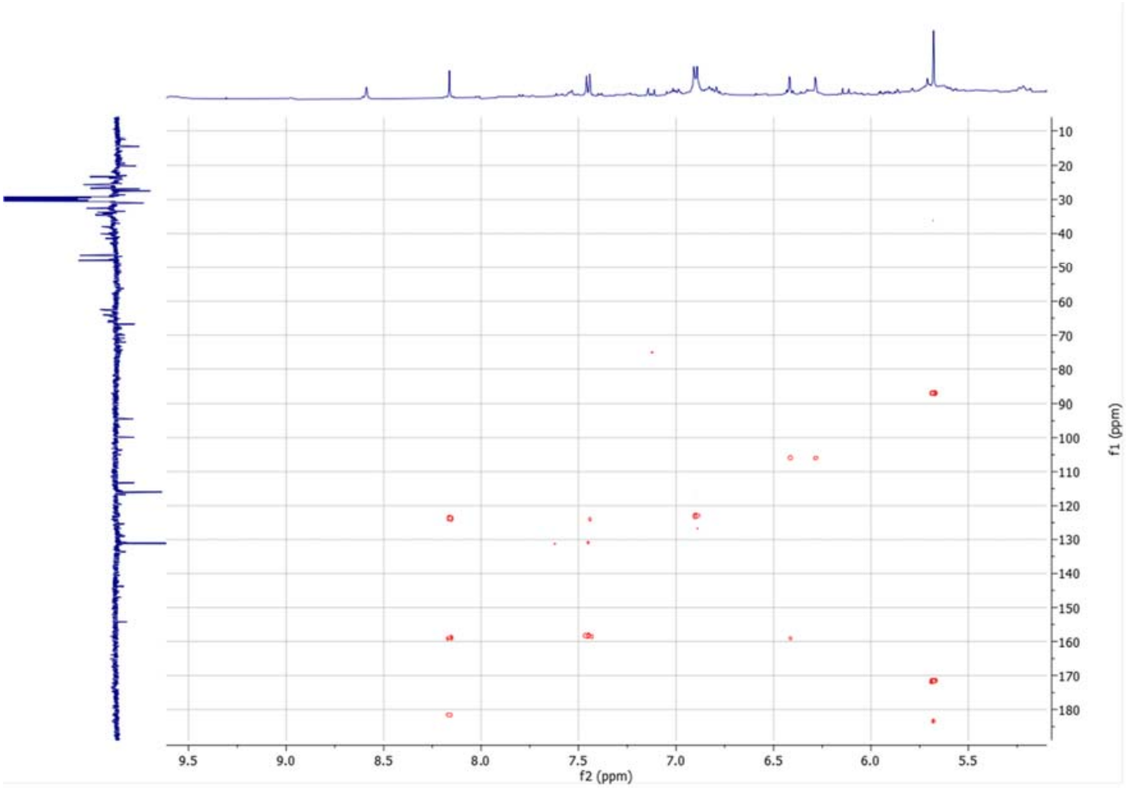

Figure S7.3. HMBC spectrum of **DL7** (Acetone–d_6)

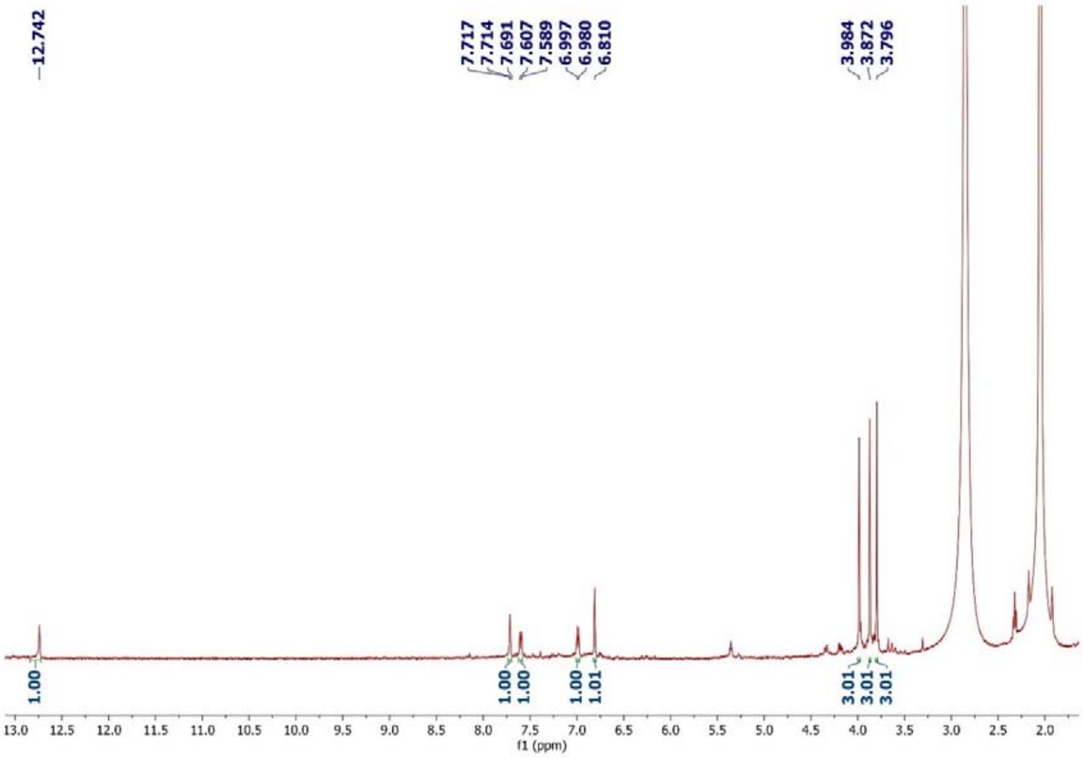

Figure S8.1. ^{1}H NMR spectrum of **DL8** (Acetone–d_6)

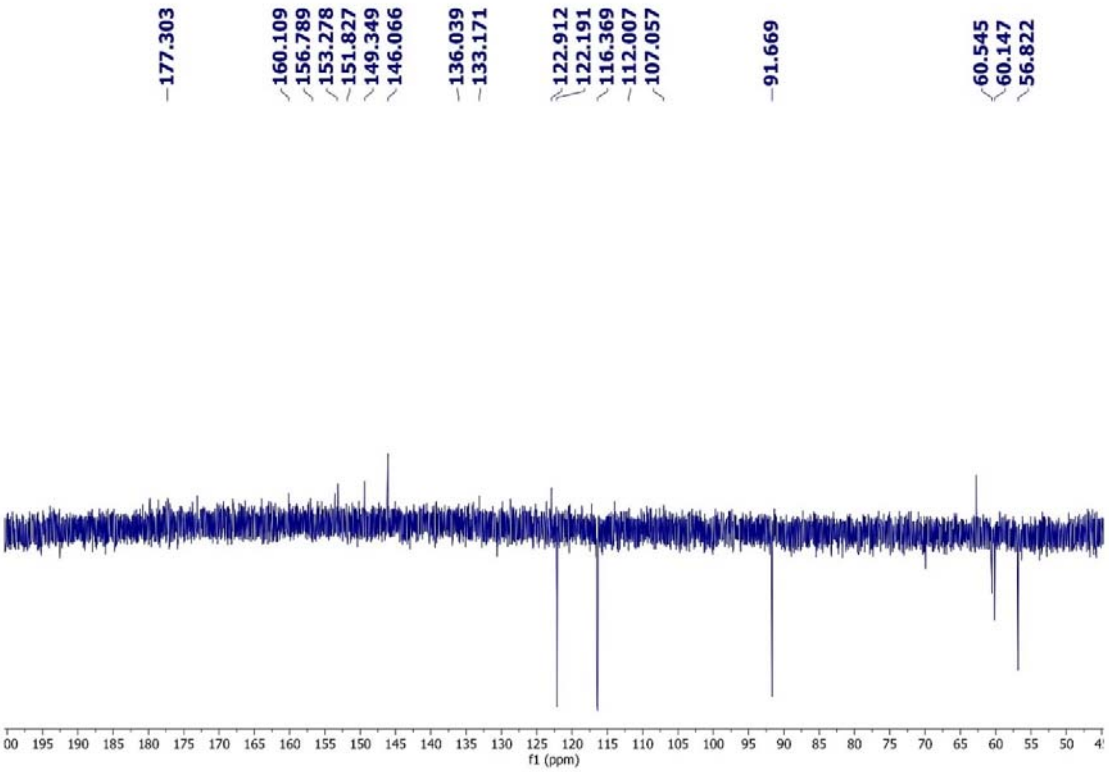

Figure S8.2. ^{13}C NMR spectrum of **DL8** (Acetone–d_6)

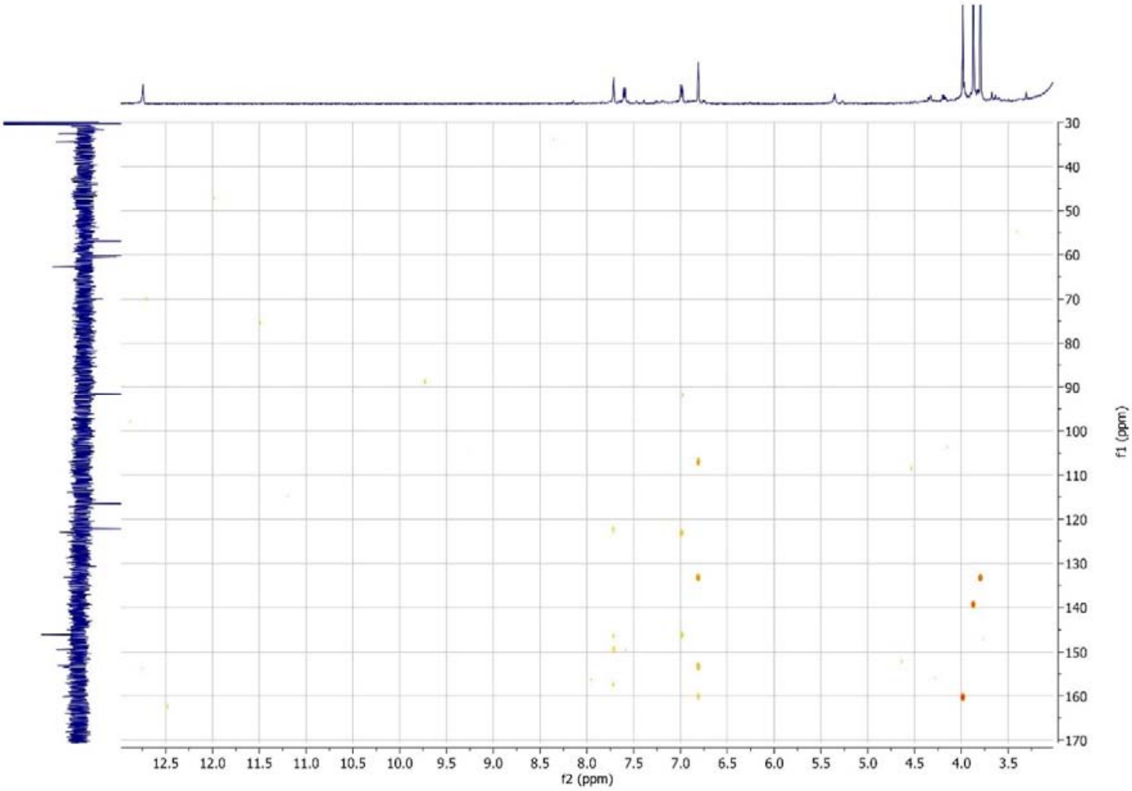

Figure S8.3. HMBC spectrum of **DL8** (Acetone–d_6)

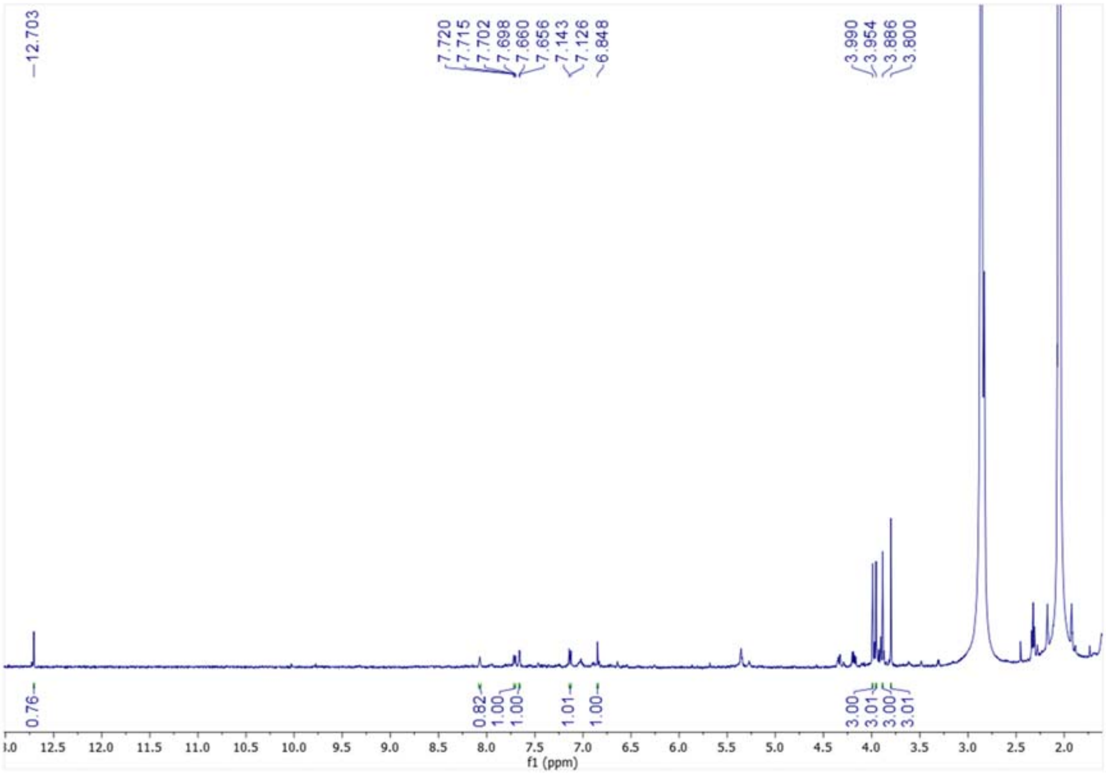

Figure S9.1. ^{1}H NMR spectrum of **DL9** (Acetone–d_6)

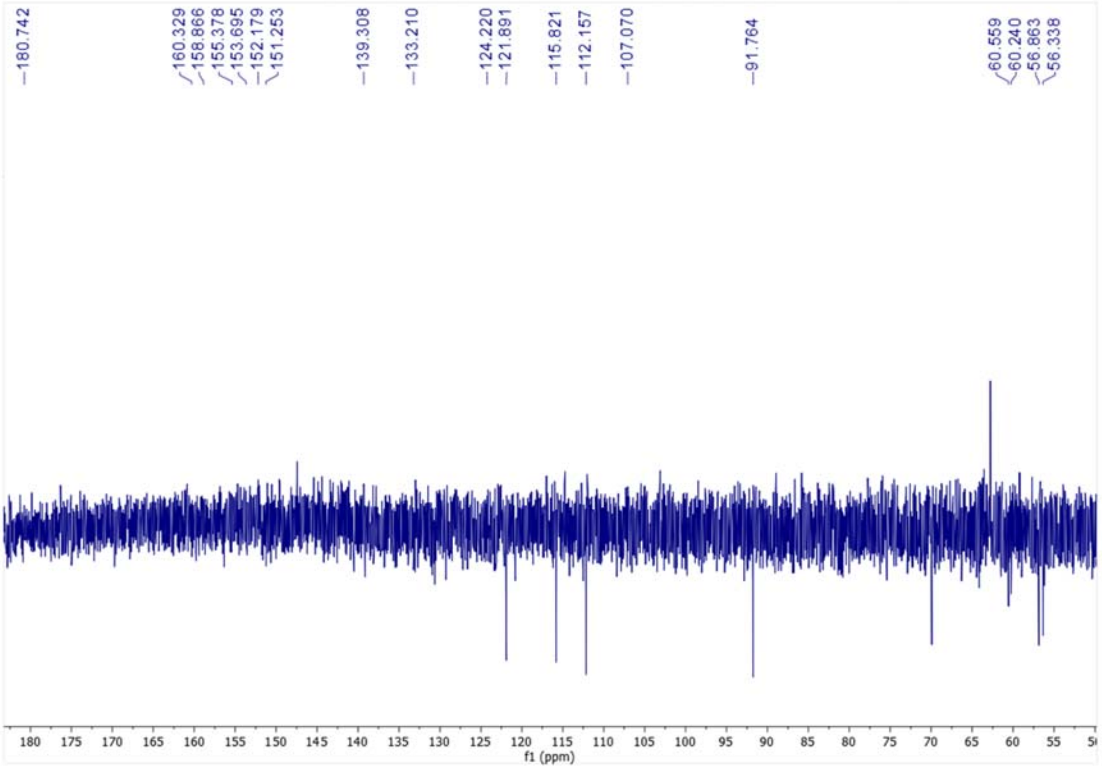

Figure S9.2. ^{13}C NMR spectrum of **DL9** (Acetone–d_6)

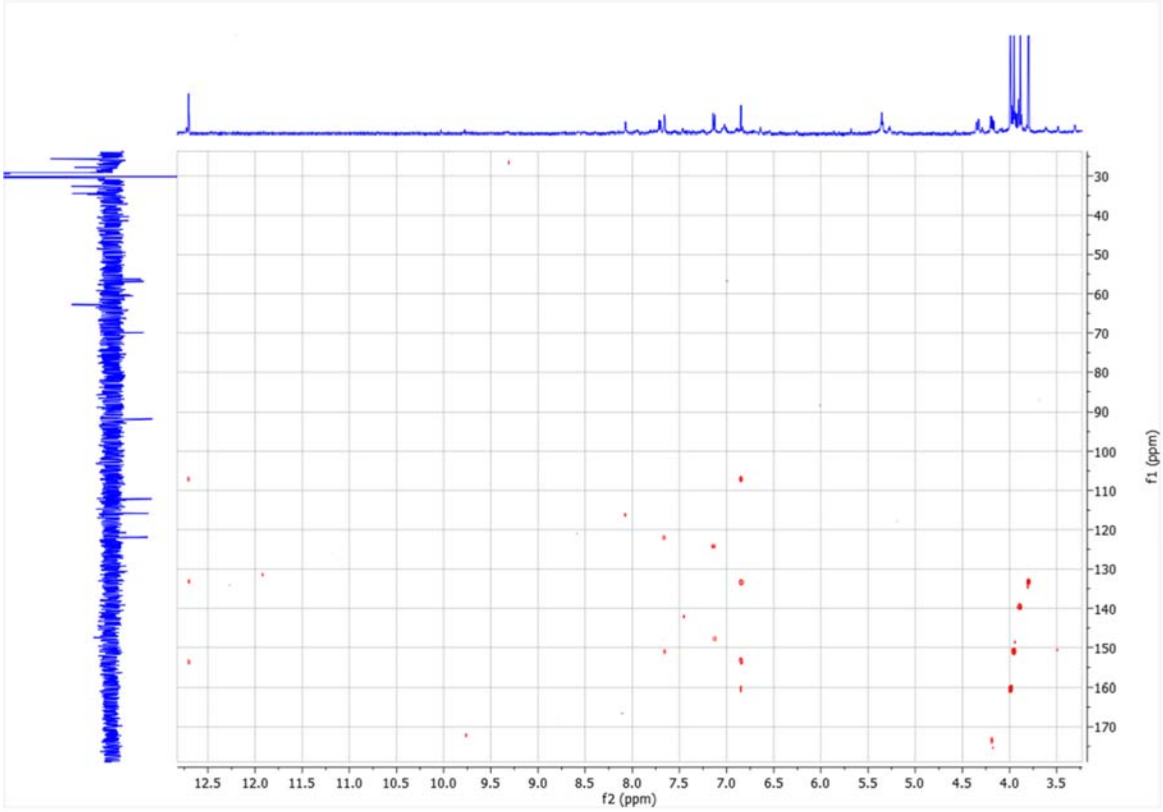

Figure S9.3. HMBC spectrum of **DL9** (Acetone–d_6)

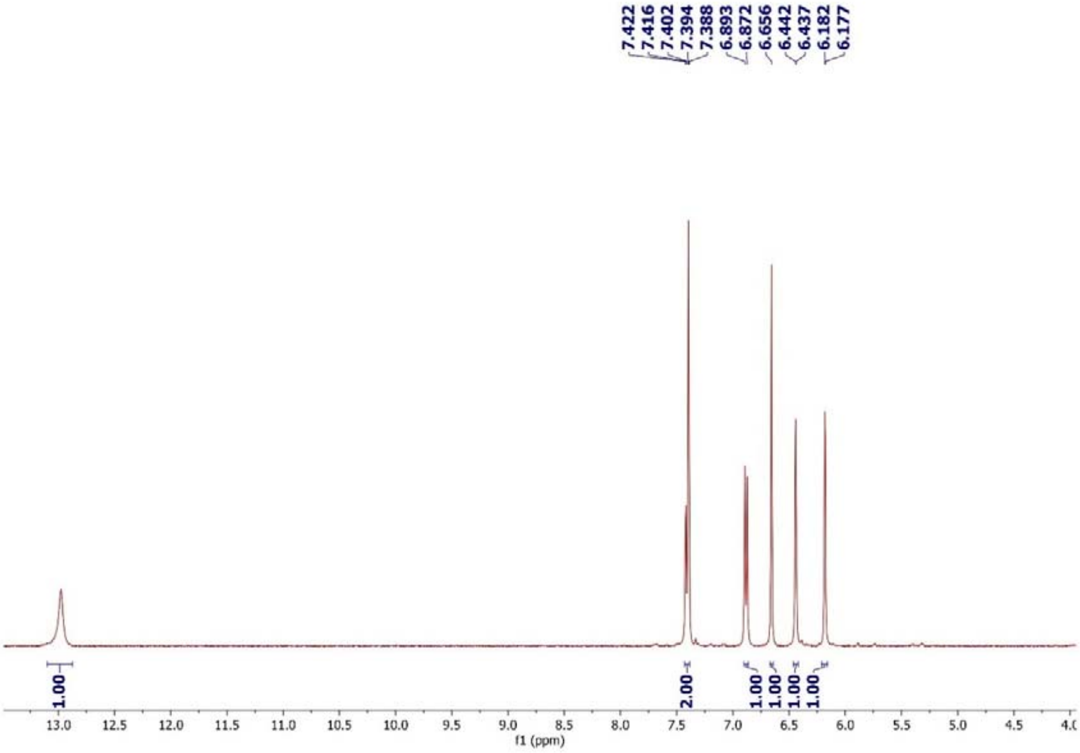

Figure S10.1. ^{1}H NMR spectrum of **DL10** (DMSO–d_6)

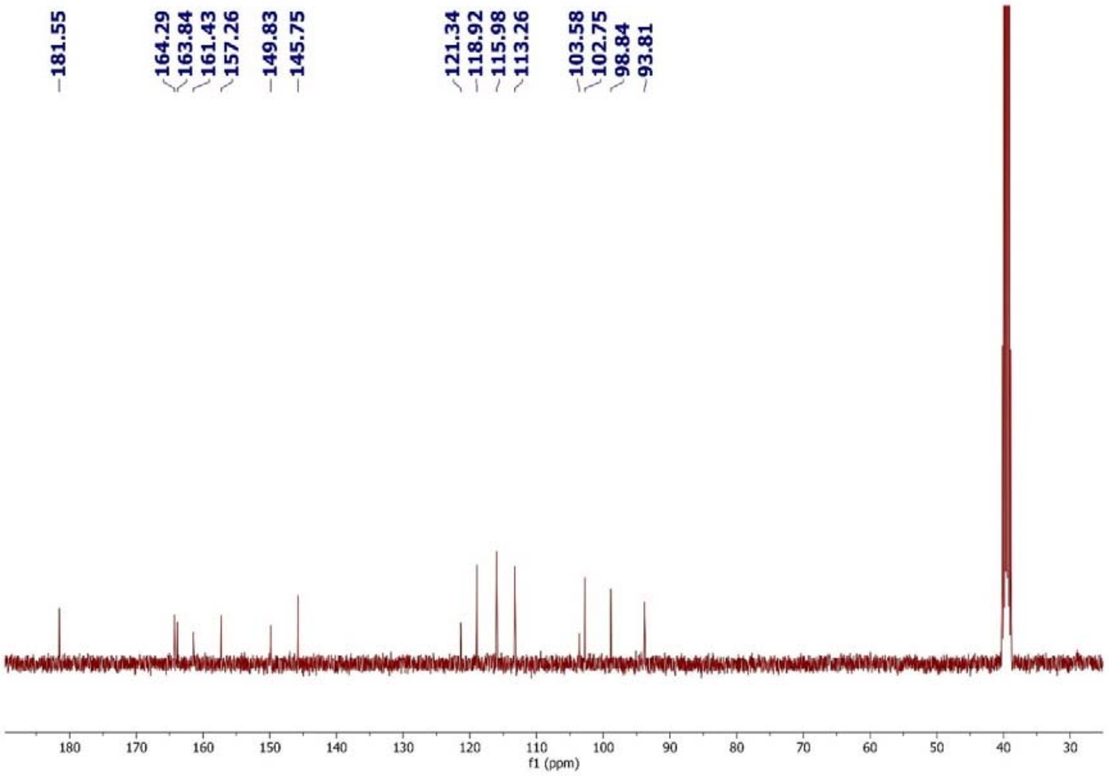

Figure S10.2. ^{13}C NMR spectrum of **DL10** (DMSO–d_6)

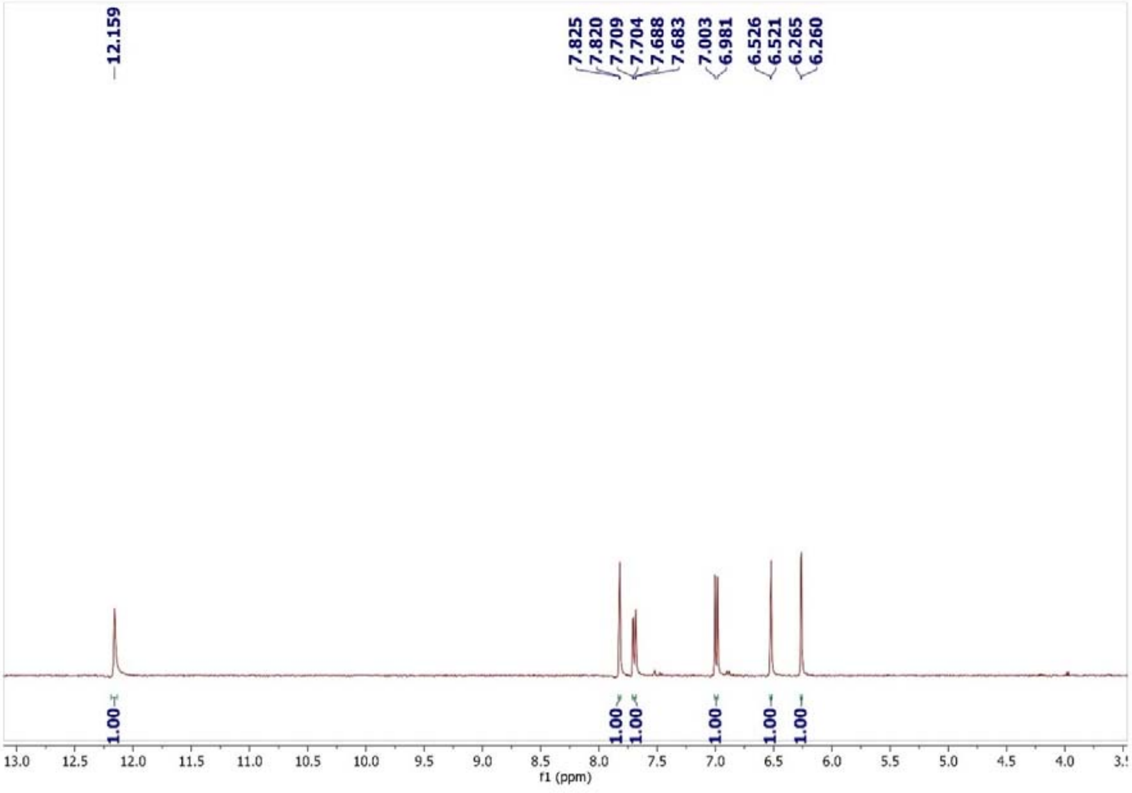

Figure S11.1. ^{1}H NMR spectrum of **DL11** (Acetone–d_6)

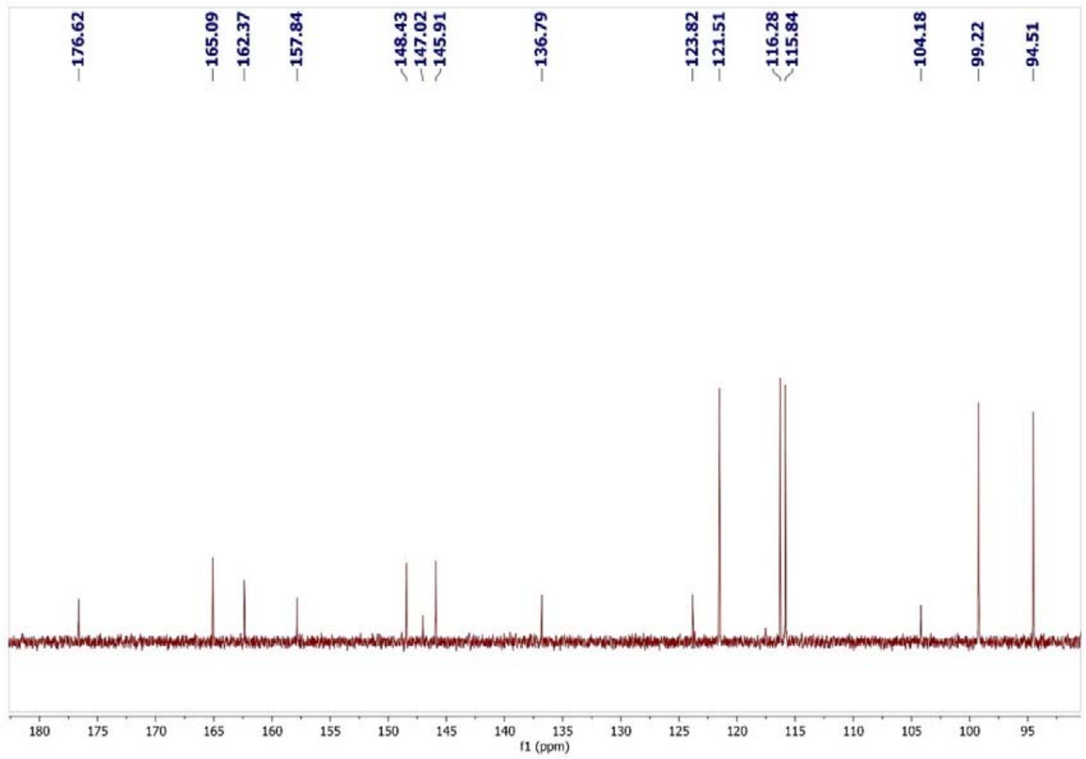

Figure S11.2. ^{13}C NMR spectrum of **DL11** (Acetone–d_6)

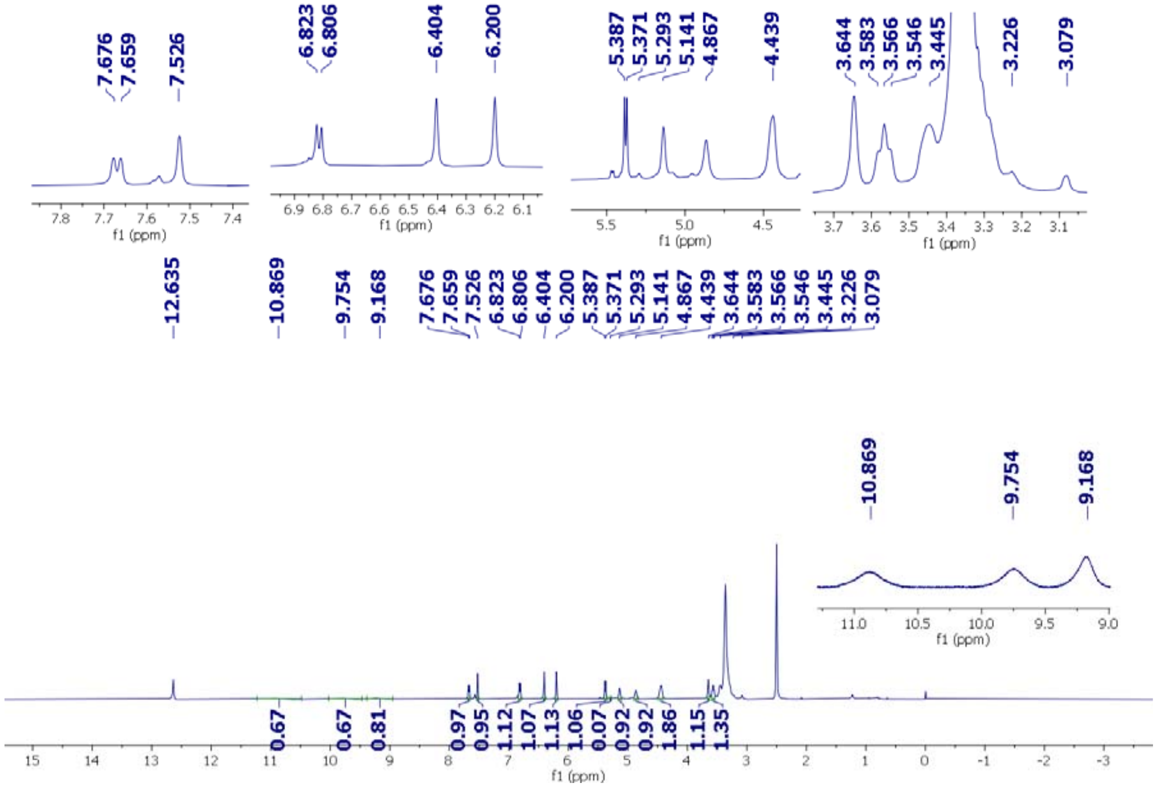

Figure S12.1. ^{1}H NMR spectrum of **DL12** (DMSO–d_6)

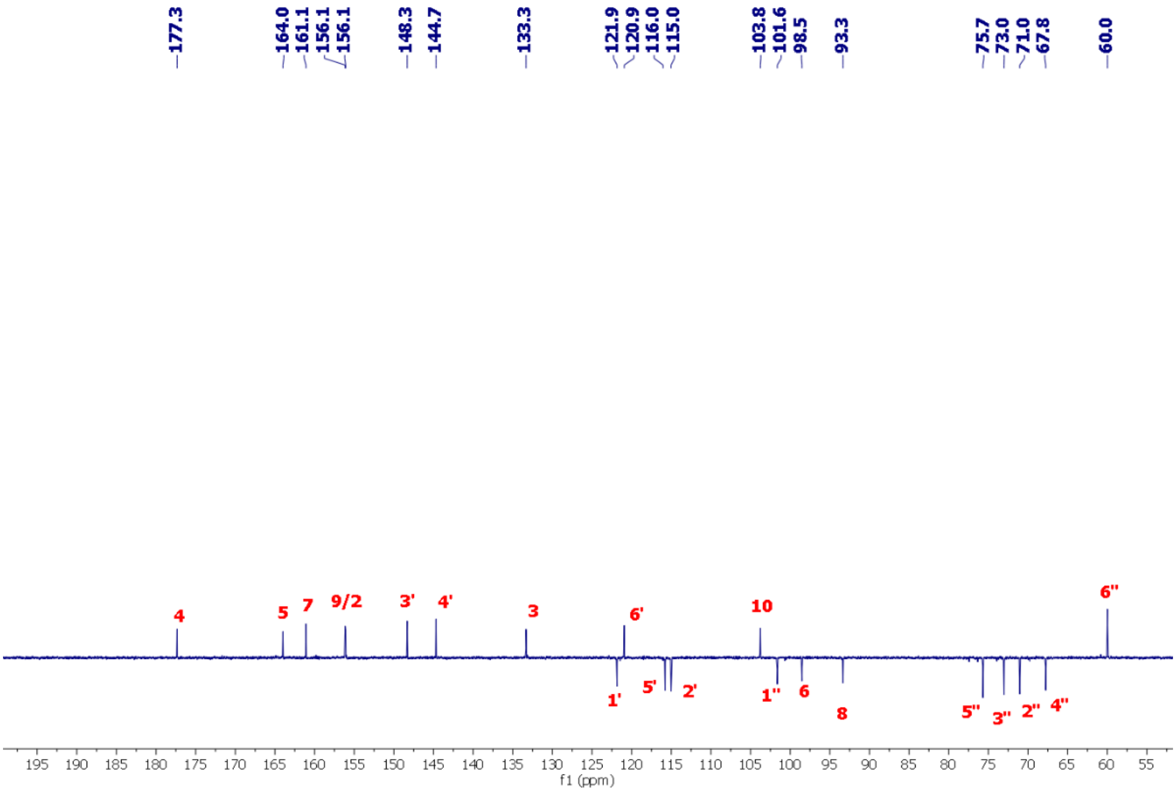

Figure S12.2. ^{13}C NMR spectrum of **DL12** (DMSO–d_6)

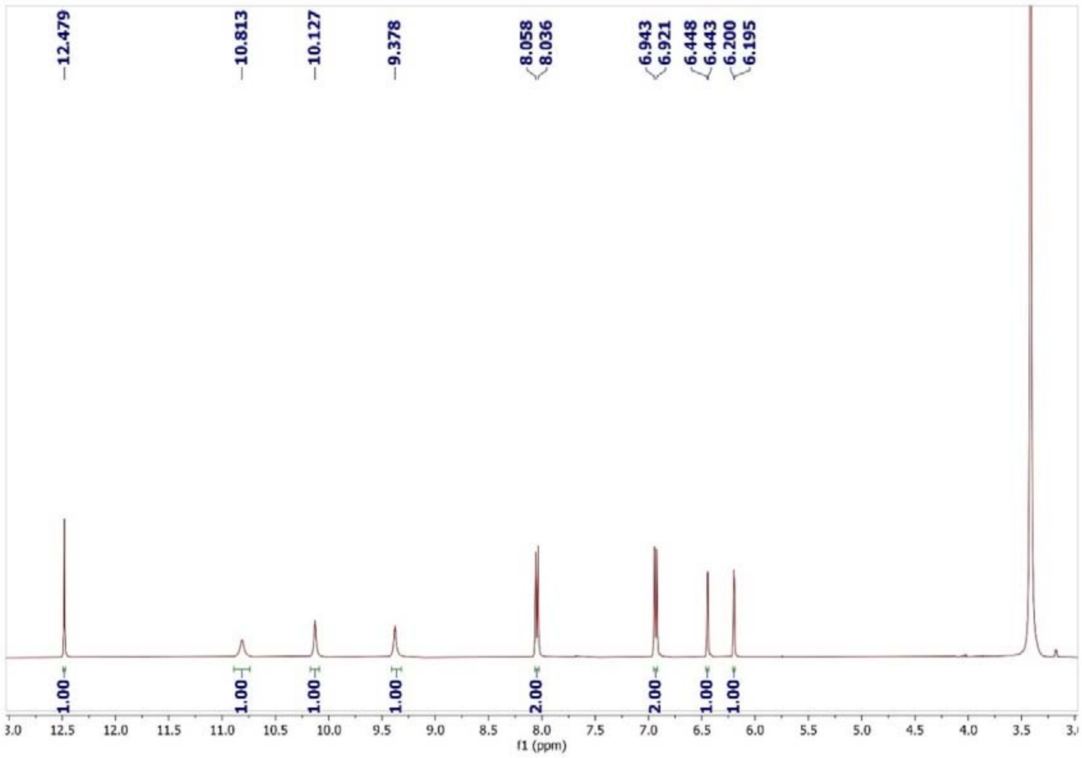

Figure S13.1. ^{1}H NMR spectrum of **DL13** (Acetone–d_6)

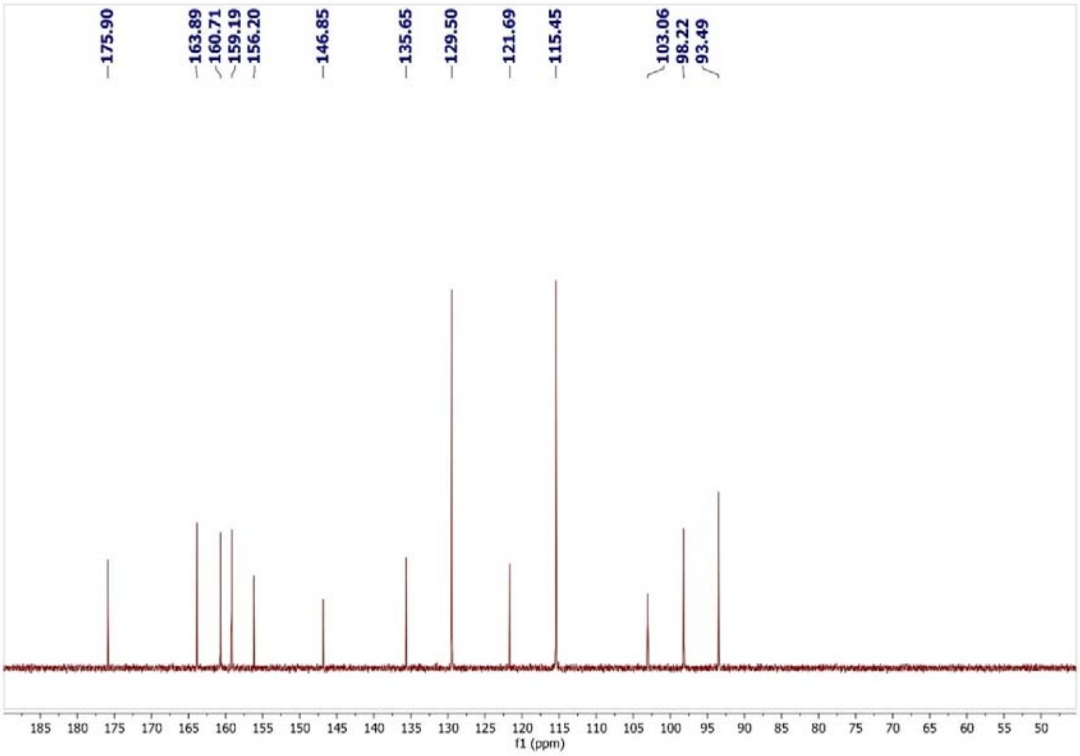

Figure S13.2. ^{13}C NMR spectrum of **DL13** (Acetone–d_6)

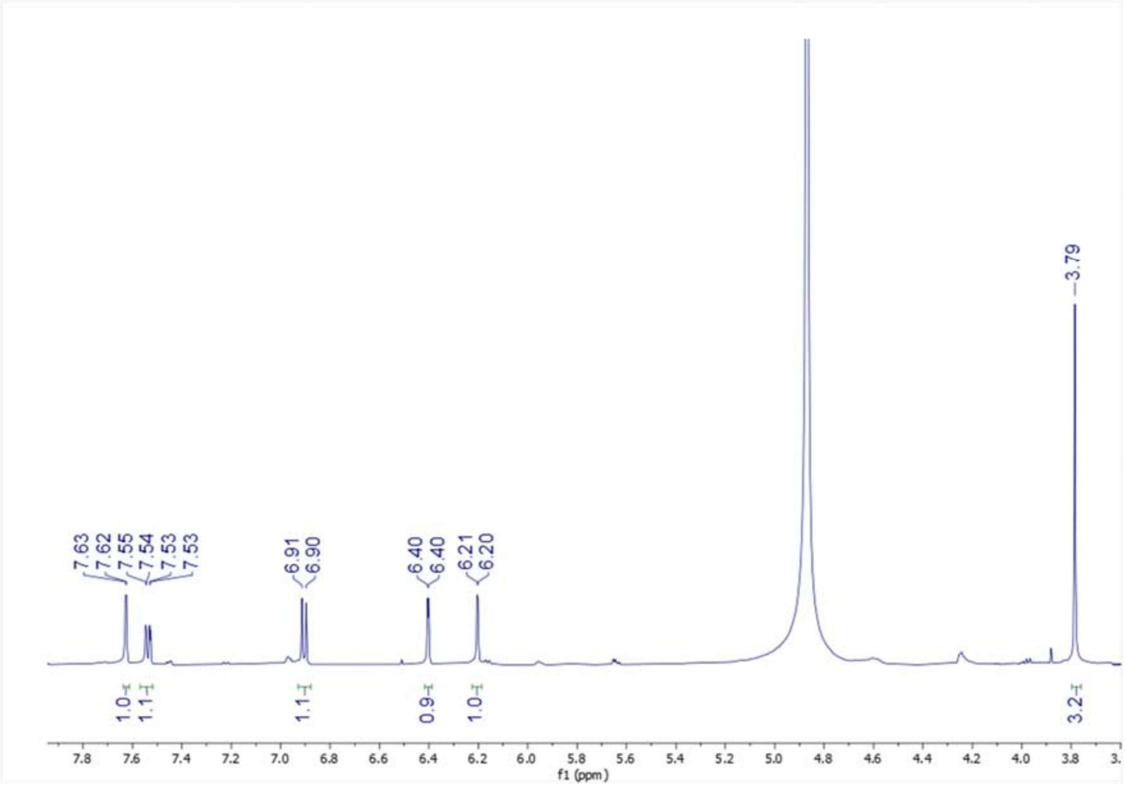

Figure S14.1. ^{1}H NMR spectrum of **PA1** (Methanol–d_4)

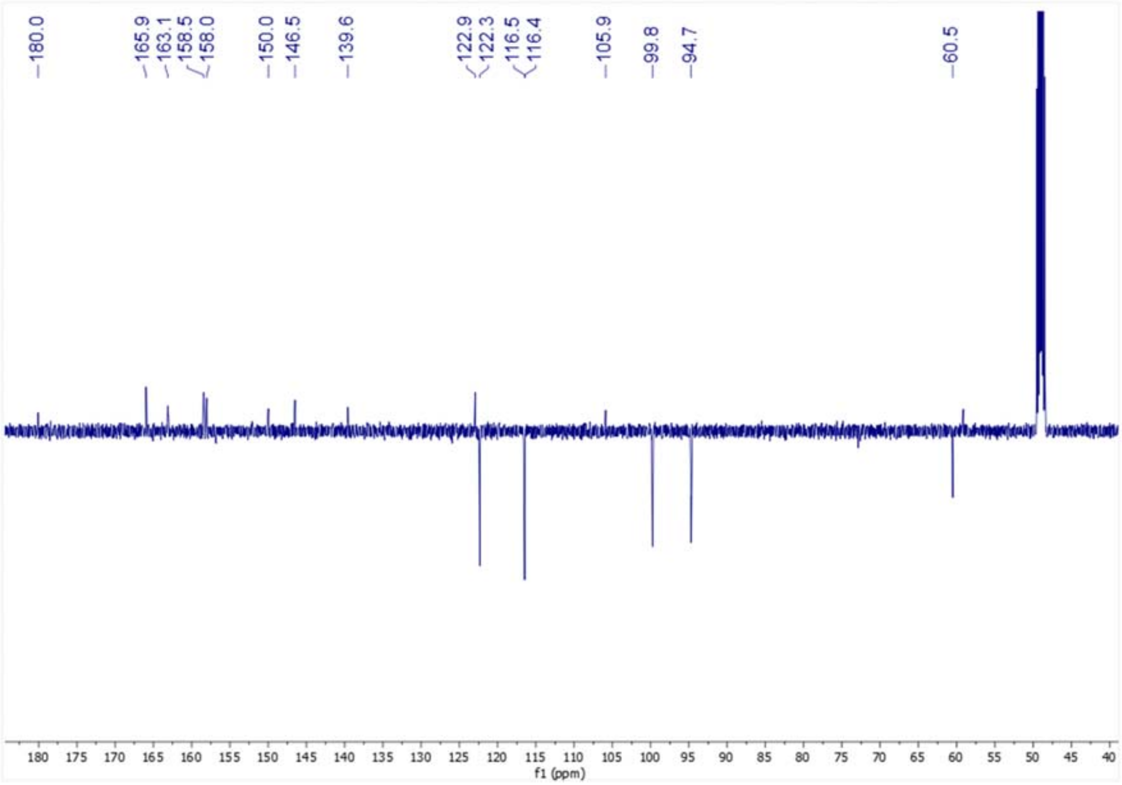

Figure S14.2. ^{13}C NMR spectrum of **PA1** (Methanol–d_4)

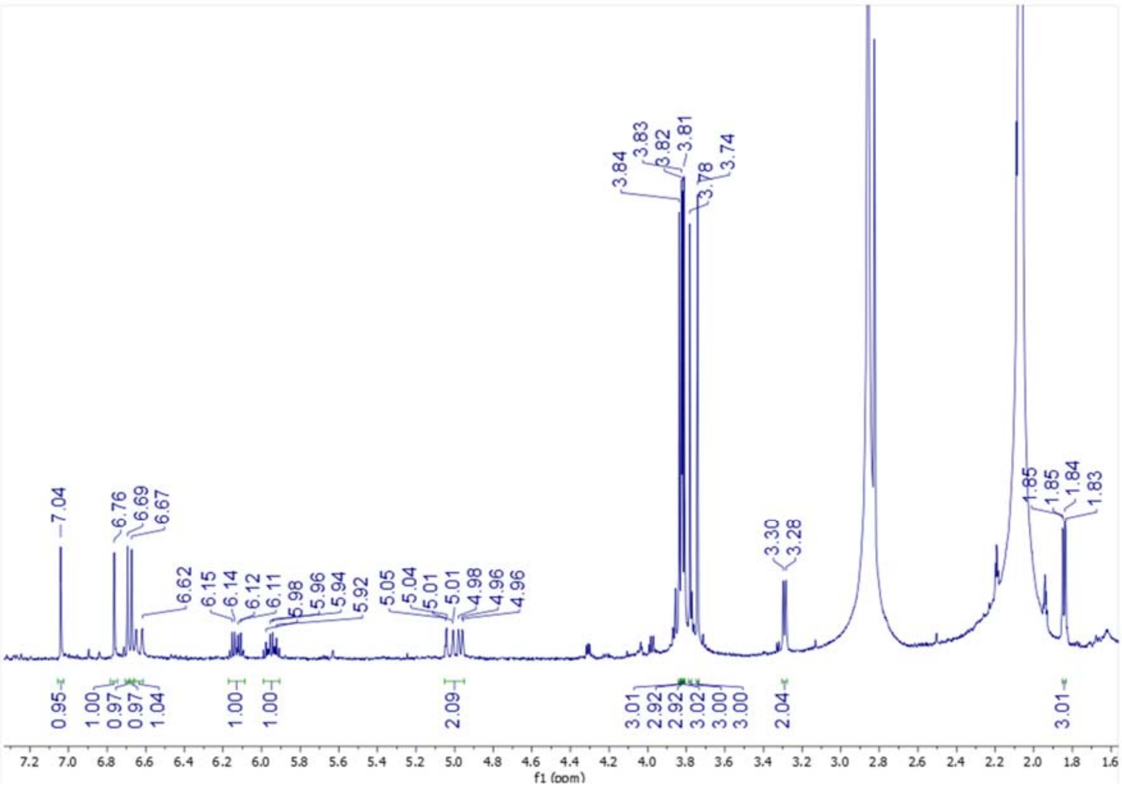

Figure S15.1. ^{1}H NMR spectrum of **PA2**and **PA3** (Acetone–d_6)

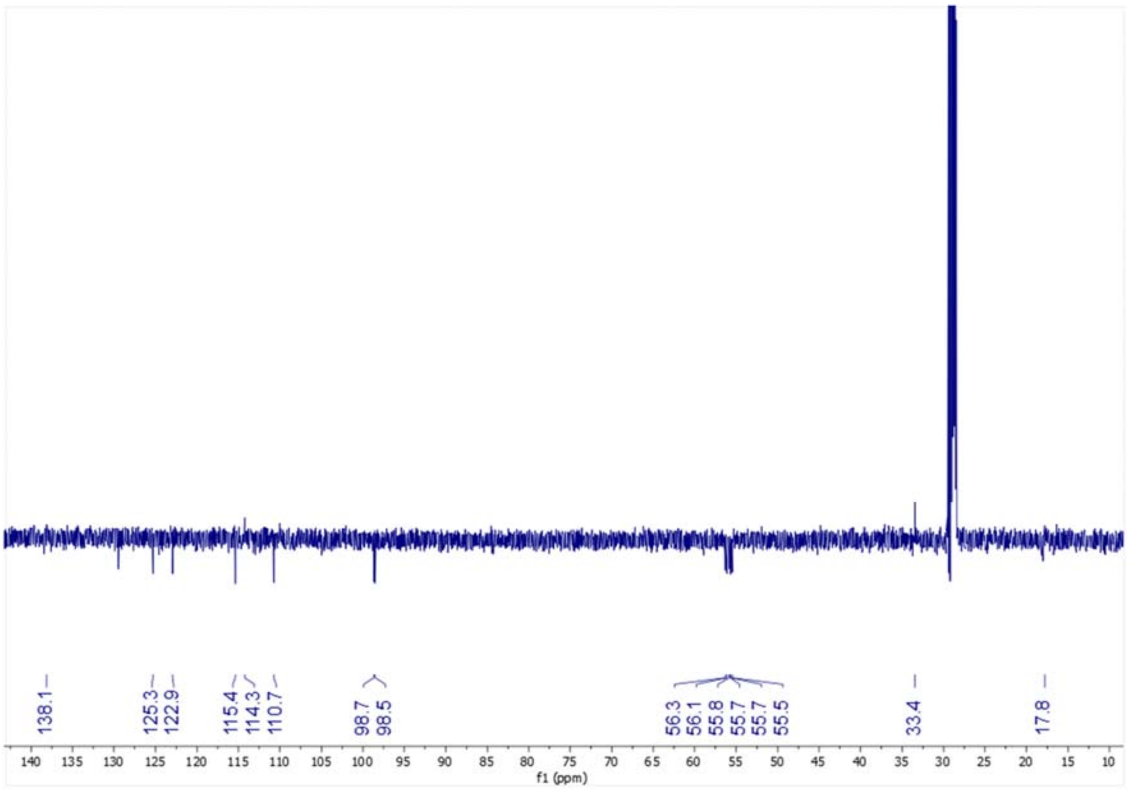

Figure S15.2. ^{13}C NMR spectrum of **PA2** and **PA3** (Acetone–d_6)

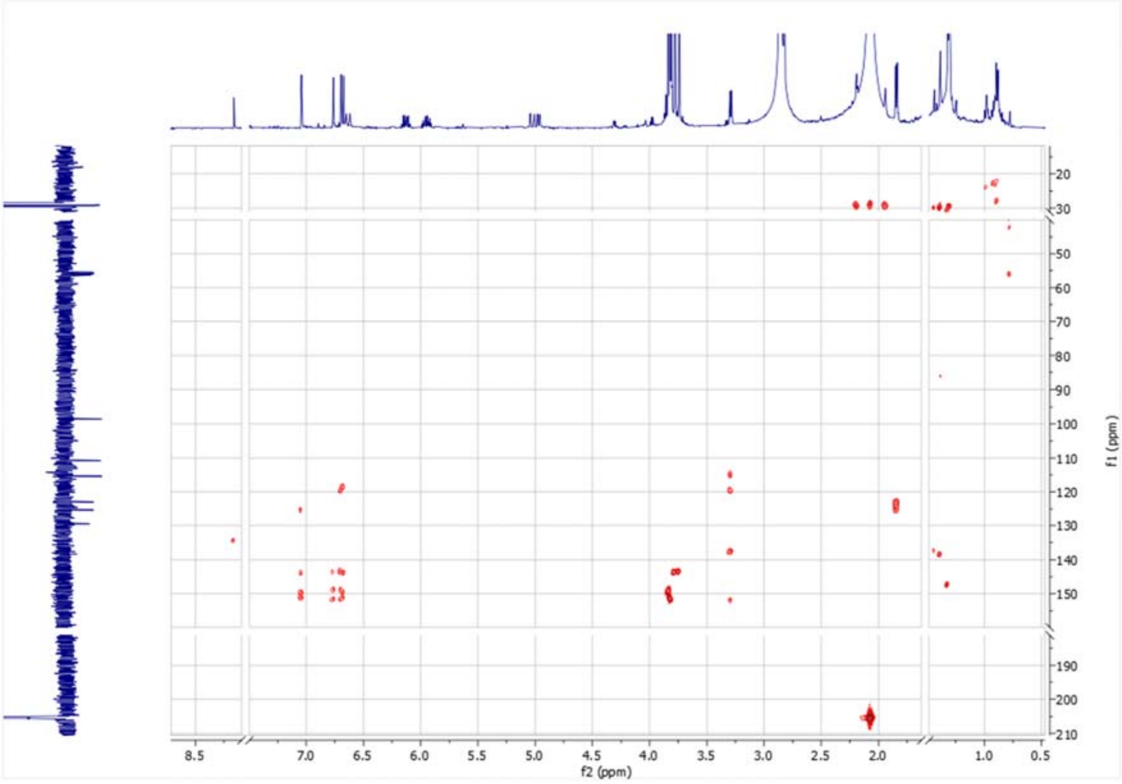

Figure S15.3. HMBC spectrum of **PA2** and **PA3** (Acetone–d_6)

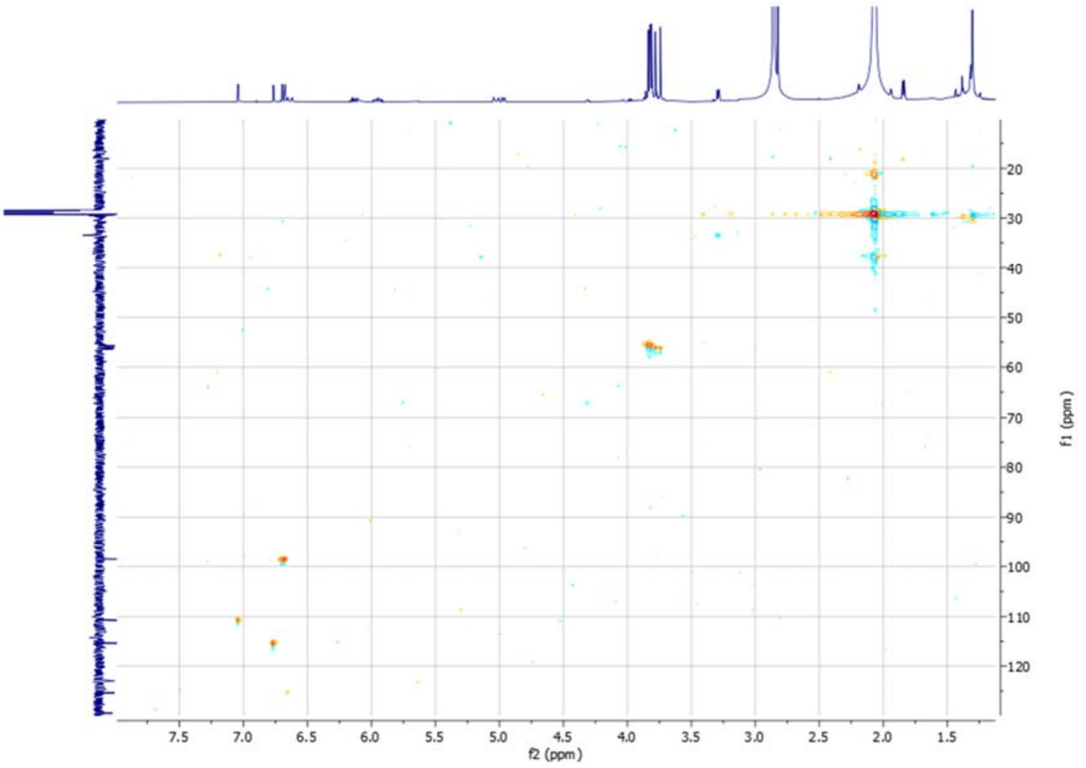

Figure S15.4. HSQC spectrum of **PA2** and **PA3** (Acetone–d_6)

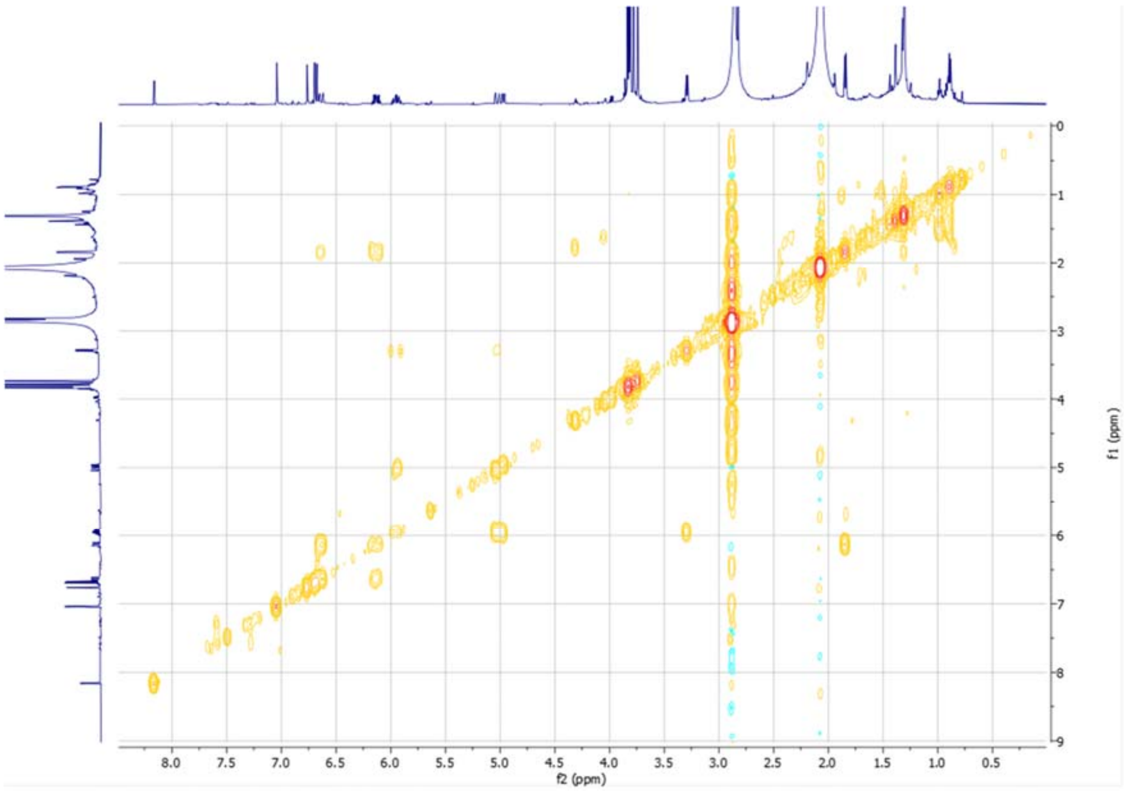

Figure S15.5. COSY spectrum of **PA2** and **PA3** (Acetone–d_6)

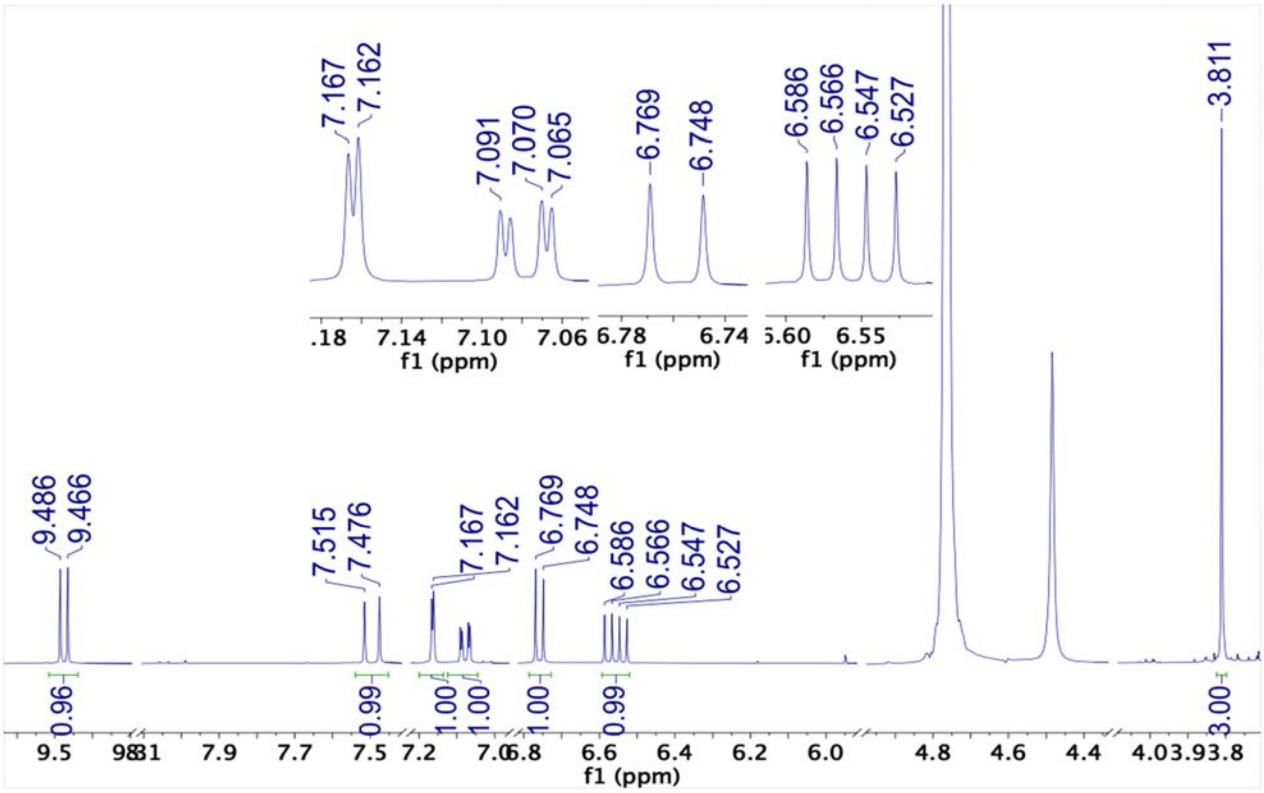

Figure S16.1. ^{1}H NMR spectrum of **PA4** (Methanol–d_4)

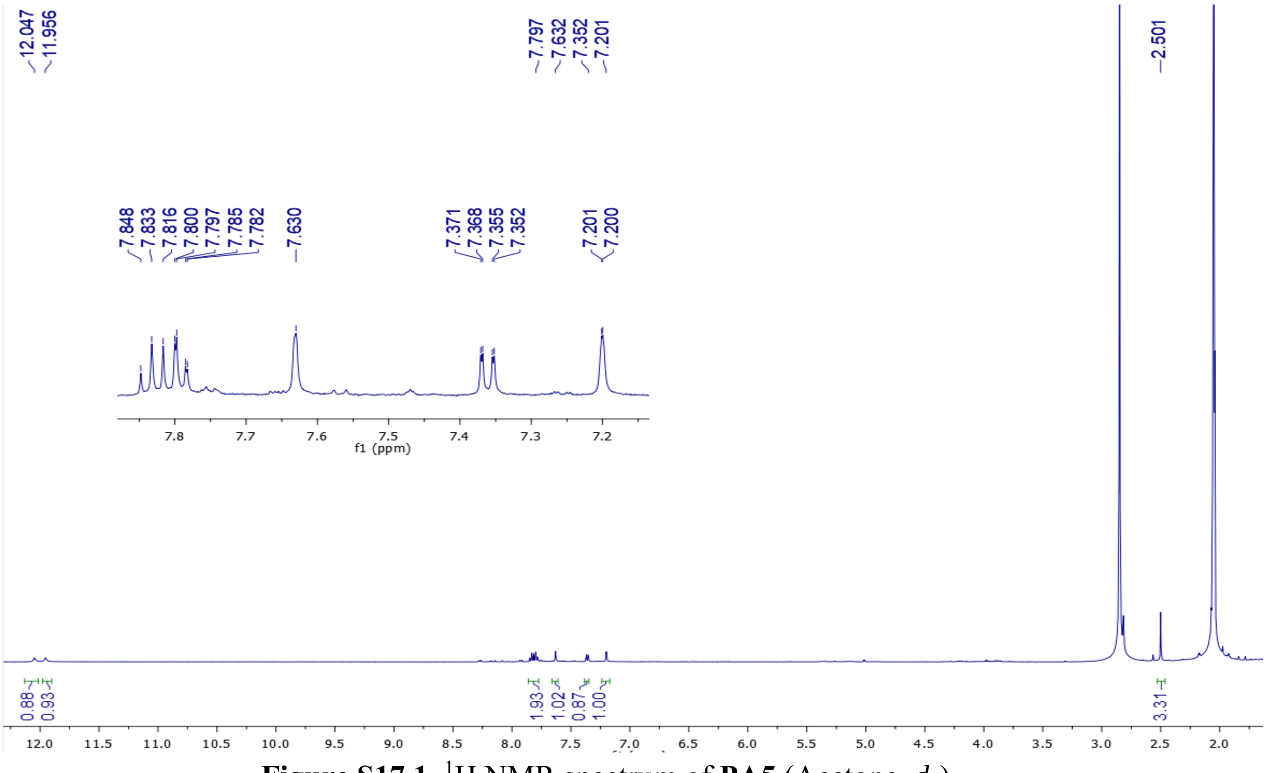

Figure S17.1. ^{1}H NMR spectrum of **PA5** (Acetone–d_6)

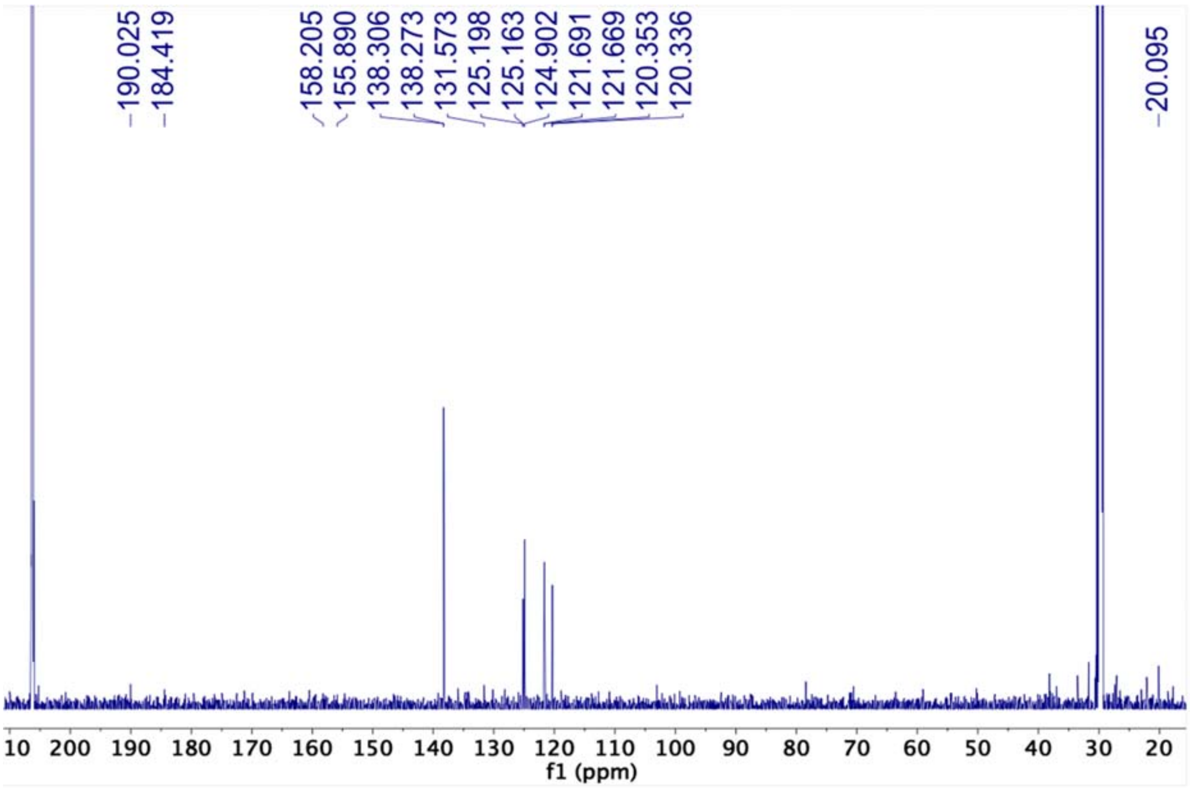

Figure S17.2. ^{13}C NMR spectrum of **PA5** (Acetone–d_6)

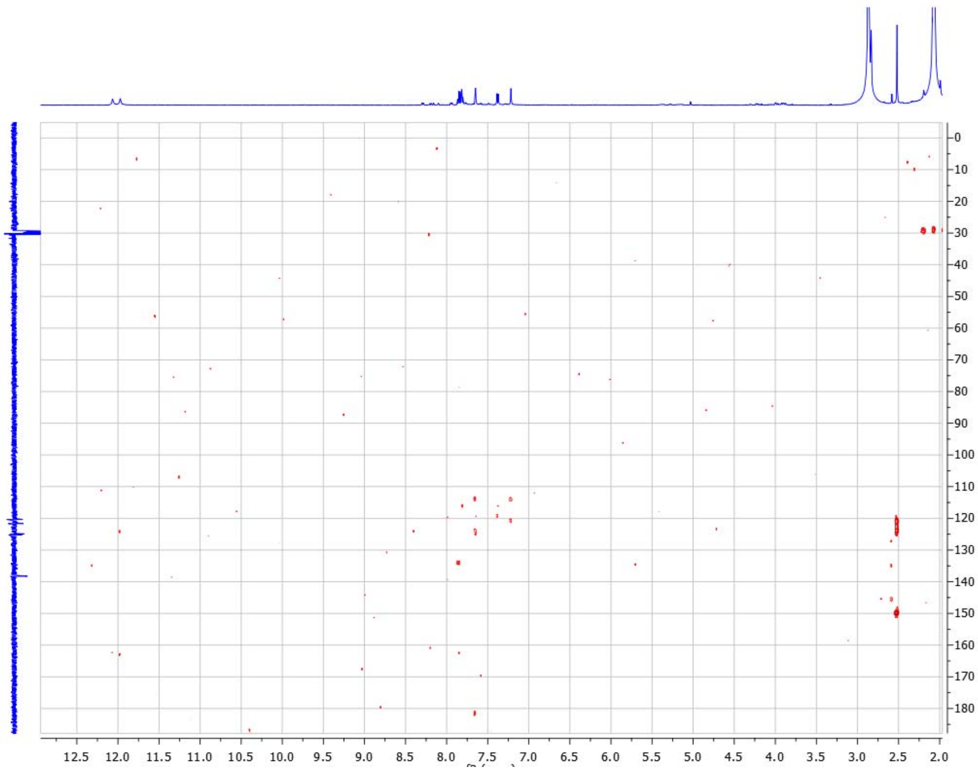

Figure S17.3. HMBC spectrum of **PA5** (Acetone–d_6)

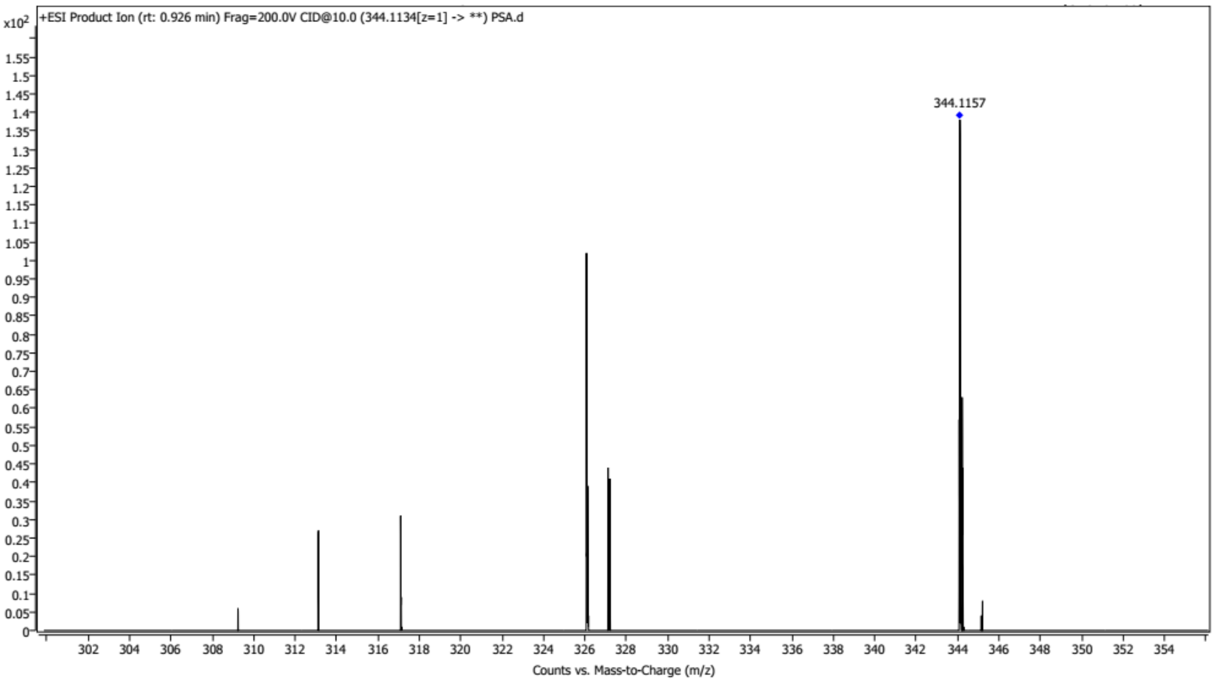

Figure S18.1. HRESIMS spectrum of **PA6** (Acetone–d_6)

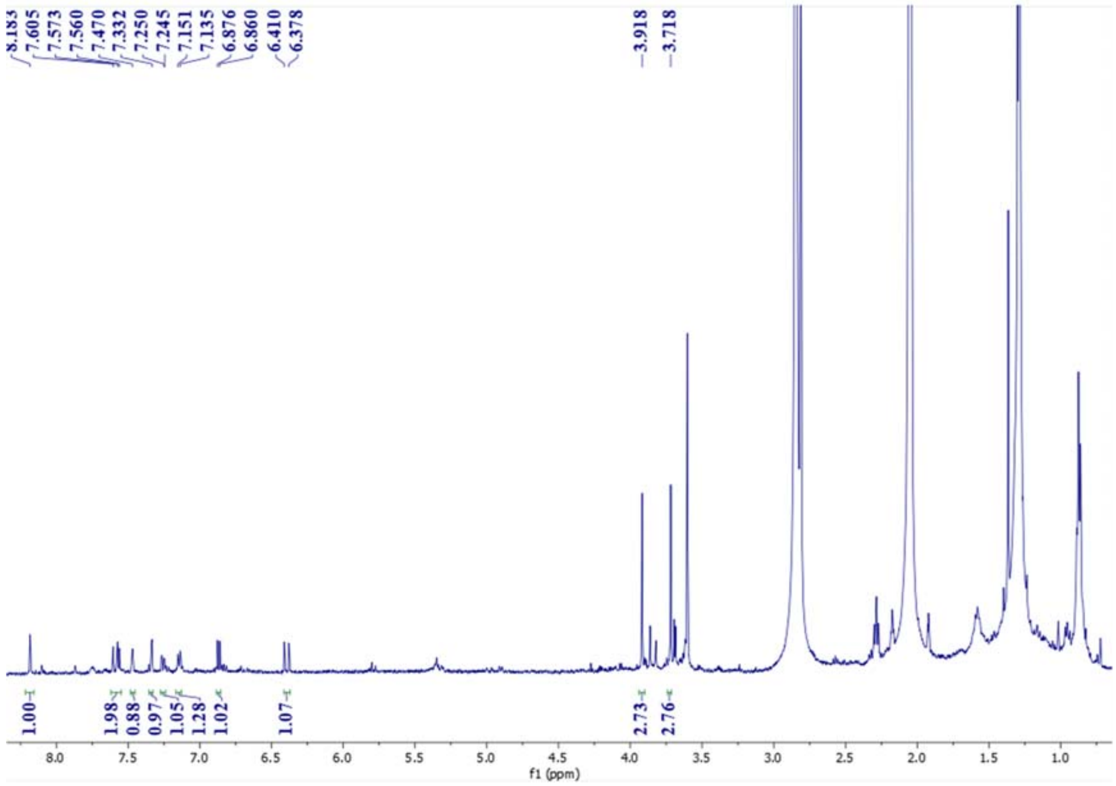

Figure S18.2. ^{1}H NMR spectrum of **PA6** (Acetone–d_6)

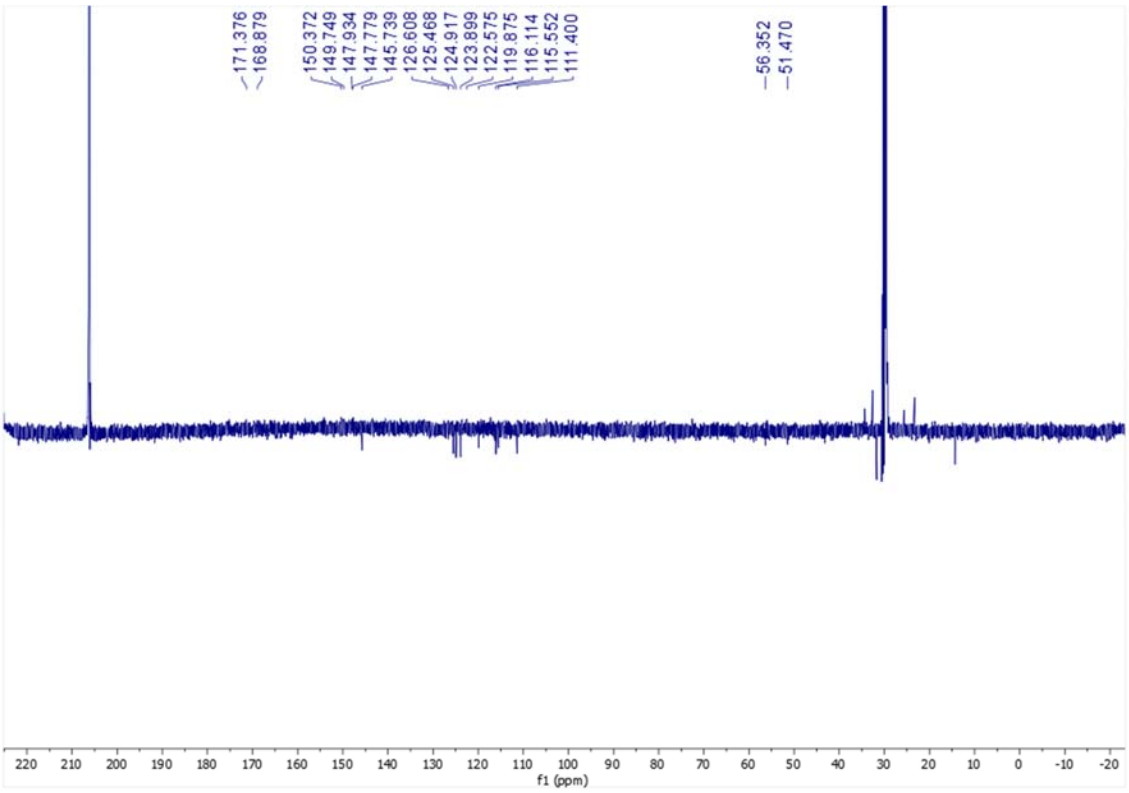

Figure S18.3. ^{13}C NMR spectrum of **PA6** (Acetone–d_6)

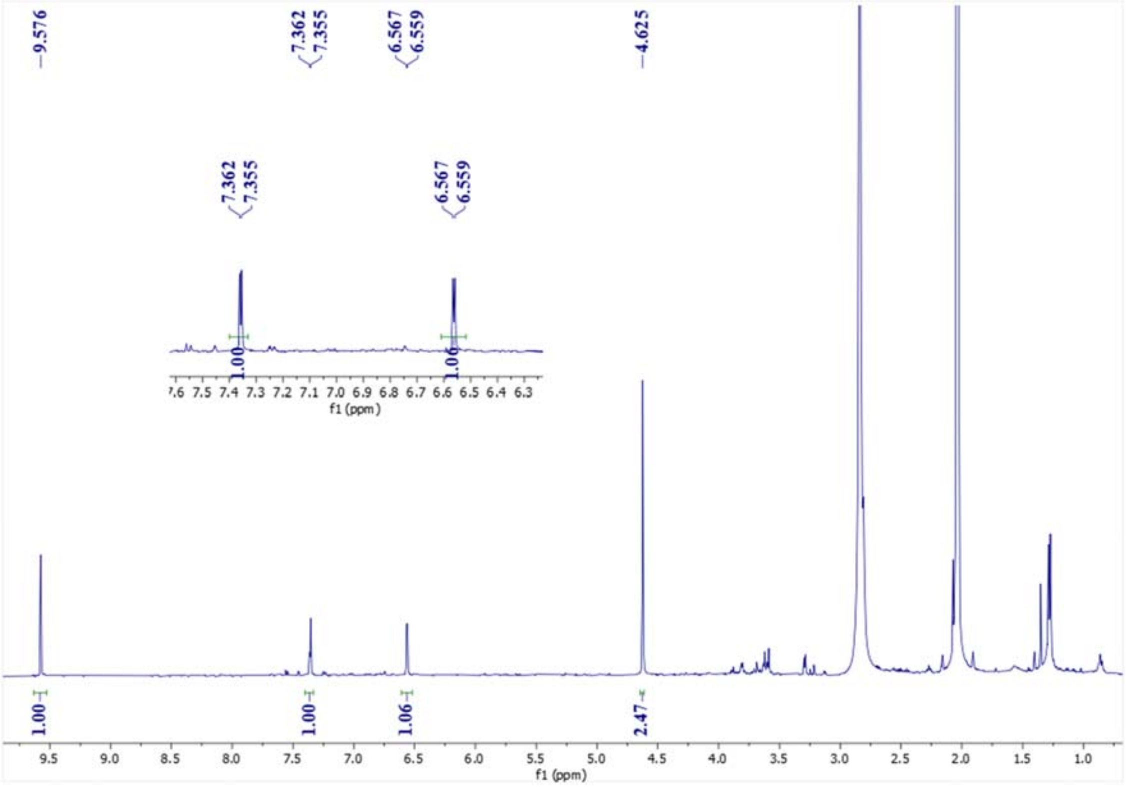

Figure S19.1. ^{1}H NMR spectrum of **PA7** (Acetone–d_6)

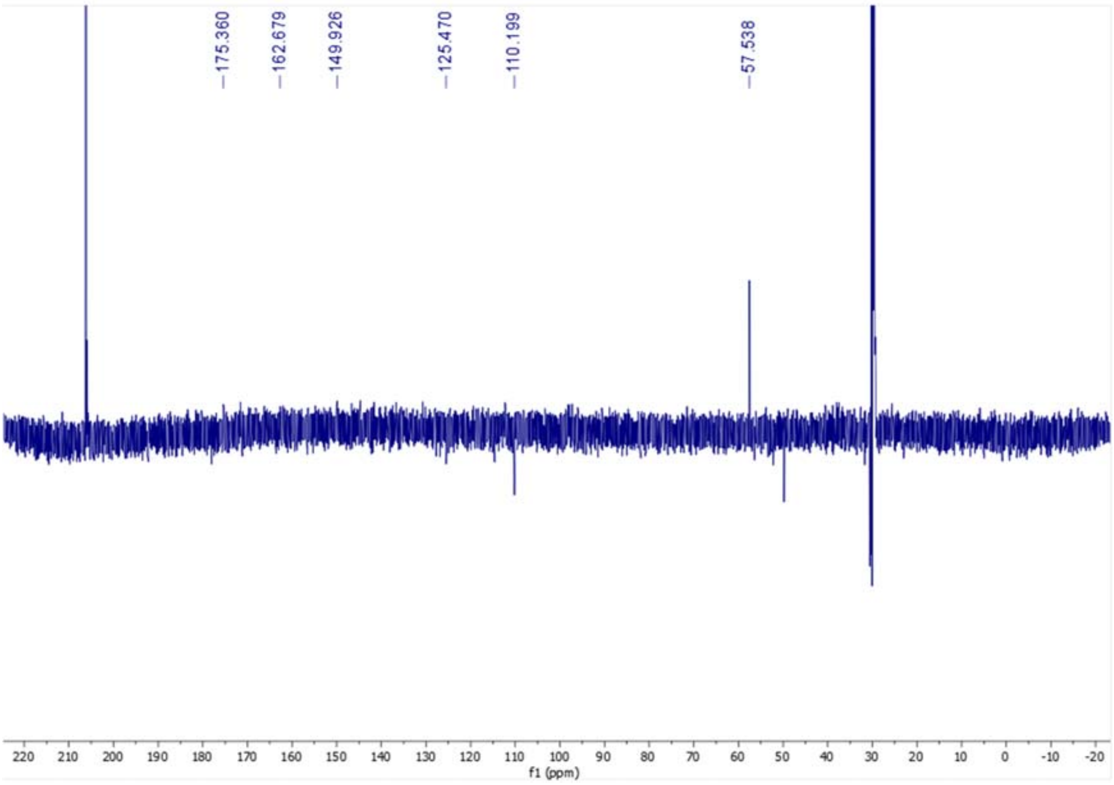

Figure S19.2. ^{13}C NMR spectrum of **PA7** (Acetone–d_6)

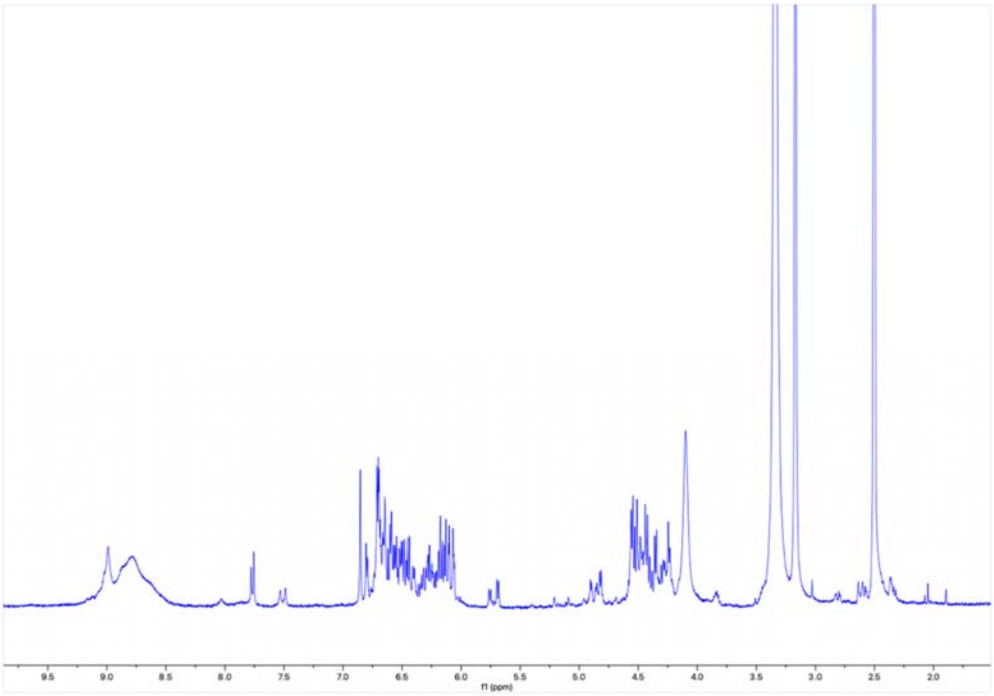

Figure S20.1. ^{1}H NMR spectrum of the mixture of lignins (Acetone–d_6)

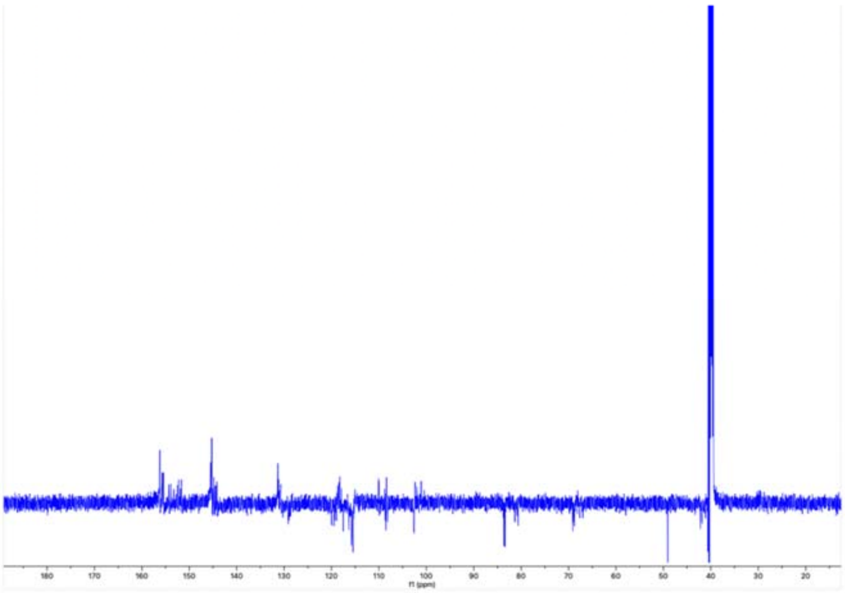

Figure S20.2. ^{13}C NMR spectrum of the mixture of lignins (Acetone–d_6)

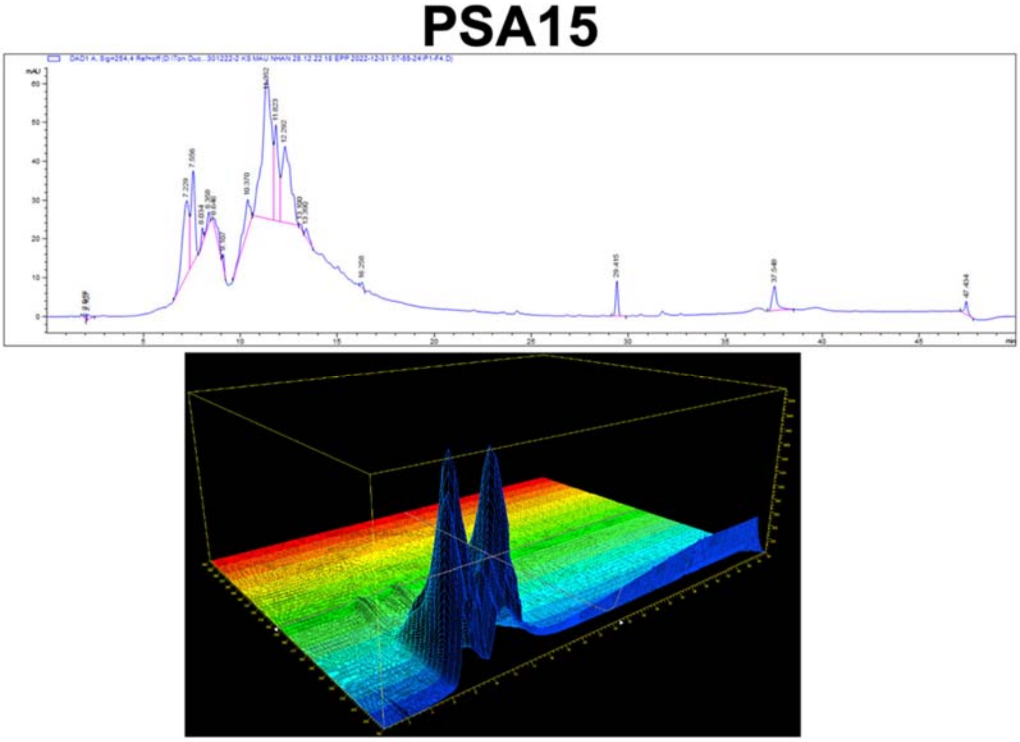

Figure S20.3 HPLC chromatogram of the mixture of lignins

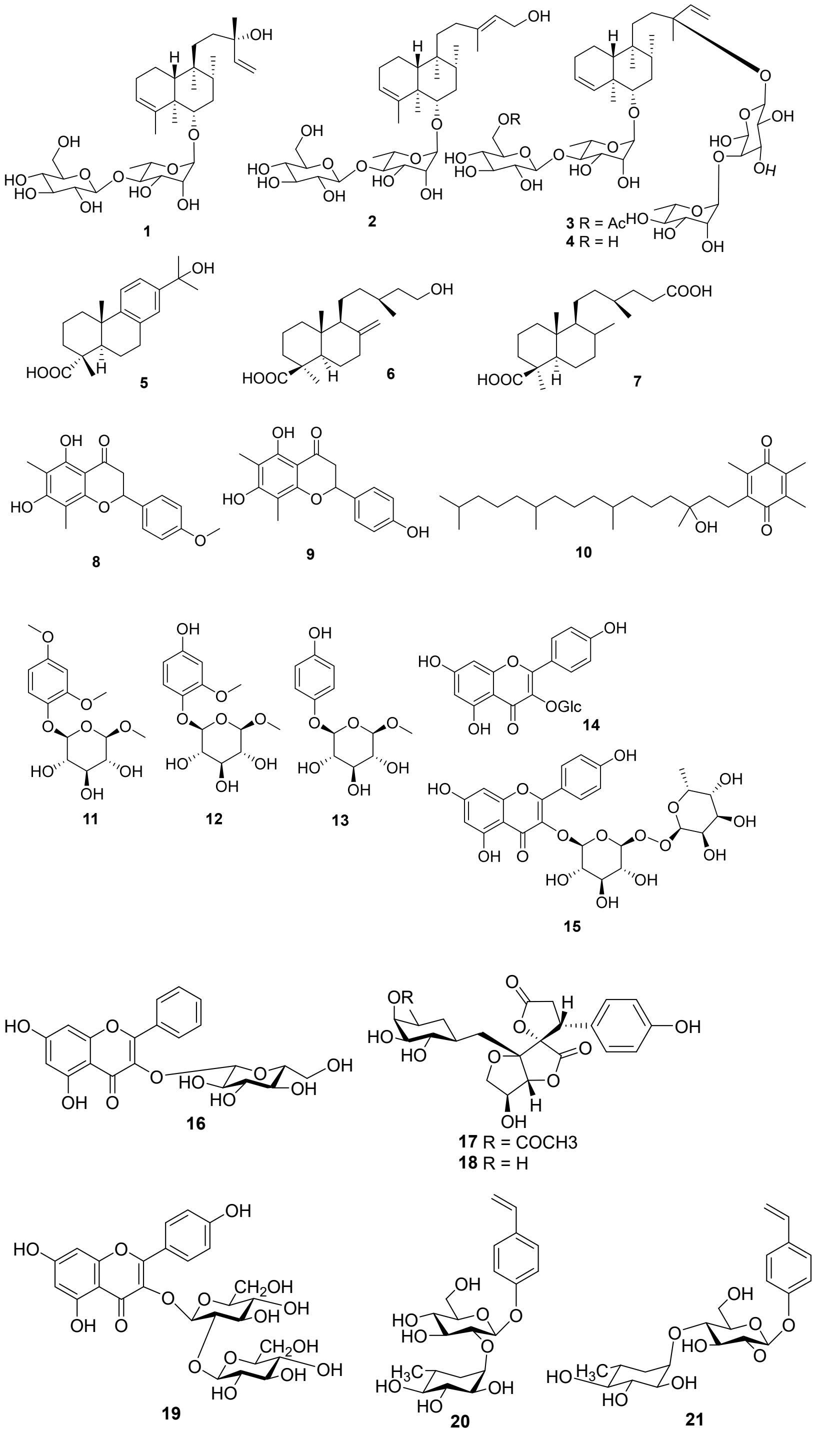

Figure S21. Chemical structures of compounds previously reported from *D. linearis*

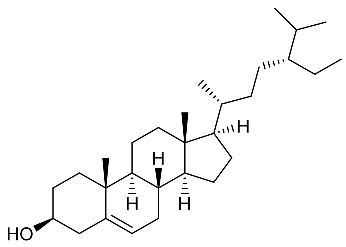

(P1)

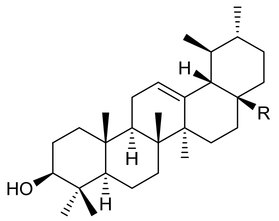

(P4) R = CH_3
(P5) R = COOH

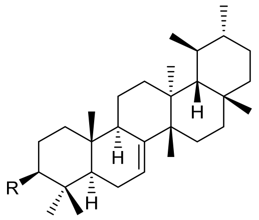

(P7) R = OH
(P8) R = AcO

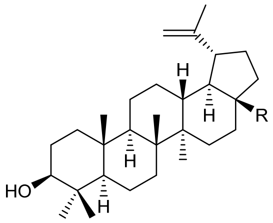

(P2) R = CH_2OH
(P3) R = COOH

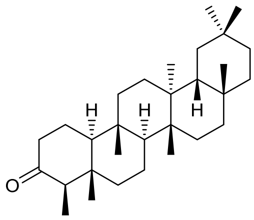

(P6)

Figure S22. Chemical structures of compounds previously reported from *P. adenophylla*
